# Lifespan‐Regulated CAR‐Macrophages from Myeloid Progenitors for Enhanced Colorectal Cancer Therapy

**DOI:** 10.1002/advs.202417677

**Published:** 2025-08-22

**Authors:** Chuancheng Gao, Fangling Hong, Yao Dong, Yong Fu, Yunong Ma, Xuedi Sun, Junfeng Zhang, Jiangning Chen, Zhen Huang

**Affiliations:** ^1^ State Key Laboratory of Pharmaceutical Biotechnology School of Life Sciences Nanjing University Nanjing Jiangsu 210023 China; ^2^ State Key Laboratory of Analytical Chemistry for Life Sciences Nanjing University Nanjing Jiangsu 210023 China; ^3^ NJU Xishan Institute of Applied Biotechnology Xishan District Wuxi Jiangsu 214101 China

**Keywords:** cancer immunotherapy, CAR‐macrophages, Myeloid progenitors, safety switch

## Abstract

Adoptive cell therapies for solid tumors face persistent challenges from poor tumor infiltration and immunosuppressive microenvironment. To overcome these limitations, a clinically scalable platform is developed to generate chimeric antigen receptor macrophages (CAR‐HMs) from tamoxifen‐regulated immortalized Hoxb8‐transduced myeloid progenitors, achieving >95% CAR transduction efficiency and 60‐fold expansion within 10 days. Engineered with a colorectal cancer‐specific anti‐carcinoembryonic antigen (CEA) CAR, these FcγRI‐CAR‐HMs demonstrated potent tumoricidal activity (>80% CRC cell lysis in vitro), deep tissue penetration (>100 µm in 3D tumor spheroids), and significant therapeutic efficacy (≈89% tumor regression in vivo). Mechanistic studies demonstrated that FcγRI‐CAR‐HMs remodeled the tumor microenvironment through direct tumor phagocytosis, T cells recruitment and activation, and synergistic enhancement of anti‐PD‐1 therapy in colorectal cancer models, while an integrated inducible caspase‐9 (iCas9) suicide switch ensured safety without compromising long‐term persistence. This progenitors‐based platform not only addresses critical manufacturing challenges but also unlocks the full therapeutic potential of CAR‐macrophages, whose unique ability to synergize with checkpoint inhibitors provides a transformative approach for treatment‐refractory solid tumors.

## Graphical abstract

Using a tamoxifen‐inducible Hoxb8 system, proliferative bone marrow progenitors can be generated, which are subsequently engineered with an anti‐CEA CAR construct containing a suicide gene (iCas9) and differentiated into CAR‐macrophages. This method facilitates scale up the production of CAR‐macrophages. Meanwhile, lifespan‐regulated CAR‐macrophages from myeloid progenitors enhance solid tumor immunotherapy, overcoming gene transduction efficiency and scalability for advanced cancer therapy.

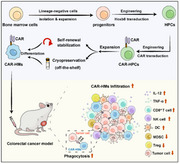

## 1 Introduction

Adoptive cell transfer therapy, notably chimeric antigen receptor (CAR) T‐cell therapy, has achieved great success in oncological treatments, particularly for hematological malignancies.^[^ ^1^, ^2^ ^]^ However, its impact on solid tumors has been less pronounced, a limitation often attributed to the complex and challenging tumor microenvironment (TME) associated with solid tumors. The TME in these cases is characterized by immunosuppressive factors, metabolic perturbations, and a dense extracellular matrix, which collectively hinder the effective infiltration and persistent activation of CAR‐T cells within the tumor tissues.^[^ ^3^, ^4^, ^5^ ^]^ Therefore, there is a critical need to explore alternative immune cell types that can effectively penetrate tumors, endure within the TME, and exert anti‐tumor effects, thereby enhancing the potential of CAR‐based therapies for solid tumor treatments.

Macrophages, in comparison to T cells, have an innate ability to penetrate tumor tissues.^[^ ^6^, ^7^, ^8^ ^]^ Their phenotypic and functional plasticity allows for sustained engagement within the TME.^[^ ^9^, ^10^, ^11^, ^12^ ^]^ Clinical evidence shows that macrophages are predominant within the TME across various types of tumors, such as colorectal cancer (CRC).^[^ ^13^, ^14^ ^]^ Beyond their phagocytic role in directly targeting tumor cells, macrophages can activate T cells through antigen presentation,^[^ ^15^, ^16^ ^]^ thereby bridging innate and adaptive immune responses against tumors. The engineering of macrophages with CARs could offer significant advantages in tumor therapy.^[^ ^17^, ^18^, ^19^, ^20^, ^21^, ^22^ ^]^ Current strategies for developing CAR‐macrophages involve the use of human THP‐1 cell lines,^[^ ^23^ ^]^ as well as macrophages derived from peripheral blood,^[^ ^24^ ^]^ cord blood, and induced pluripotent stem cells (iPSCs).^[^ ^25^, ^26^, ^27^ ^]^ However, cell lines are not considered clinically viable, and macrophages differentiated from peripheral blood mononuclear cells and umbilical cord blood encounter issues such as limited availability, donor variability, contamination risk, and exhaustion post‐CAR modification.^[^ ^28^, ^29^, ^30^ ^]^ Additionally, terminally differentiated macrophages are challenging to expand and typically exhibit low gene transduction efficiency.^[^ ^25^ ^]^ Consequently, researchers have explored the use of CD34^+^ hematopoietic stem and progenitor cells (HSPCs) to induce macrophage differentiation; however, these cells are limited by their low proliferative capacity and a propensity for premature differentiation.^[^ ^31^, ^32^, ^33^ ^]^ Moreover, chemotherapy‐induced bone marrow suppression in cancer patients often requires the repeated mobilization and collection of HSPCs.^[^ ^34^, ^35^ ^]^ These factors collectively present significant barriers to generating large, high‐purity populations of CAR‐macrophages suitable for clinical applications. iPSCs‐derived CAR‐macrophages offer a promising alternative due to their self‐renewal capabilities and scalability. While CD34^+^ HSPCs can be reprogrammed into iPSCs and then differentiated into macrophages for CAR‐macrophage production, the process remains time‐consuming.^[^ ^25^, ^27^ ^]^ Therefore, the identification of new progenitor types that possess self‐renewal and expansion potential, shorter induction times, and high differentiation efficiency into macrophages is critical for advancing CAR‐macrophage therapy development.

In this study, we developed a three‐step strategy for the genetic engineering of CAR macrophages derived from myeloid progenitors. Initially, we employed a retroviral vector carrying the tamoxifen‐inducible Hoxb8 gene to generate proliferative bone marrow progenitors, designated as Hoxb8 progenitors (HPCs). Subsequently, a lentivirus vector encoding the anti‐carcinoembryonic antigen (CEA)‐CAR, specific for a colorectal cancer antigen, was transduced into HPCs to establish a single‐cell clone of CAR‐HPCs with self‐renewal capabilities. Finally, we leveraged the differentiation potential of CAR‐HPCs to develop into CAR‐macrophages. This progenitor‐based genetic modification approach effectively addresses the low gene transduction efficiency and exhaustion typically associated with primary macrophages. The monoclonal CAR‐HPC clone could be rapidly expanded to yield a substantial quantity of macrophages that consistently express typical macrophage markers and elevated levels of CARs. Cellular studies demonstrate that CAR‐mediated activation of HPCs significantly enhances macrophage phagocytic and tumoricidal activities against colorectal cancer cells. CAR‐macrophages were found to efficiently infiltrate tumor tissues and effectively reverse the immunosuppressive TME. Moreover, when combined with immune checkpoint inhibitors, CAR‐macrophages effectively inhibit tumor growth. The inclusion of the suicide gene inducible caspase‐9 (iCas9) allows for precise in vivo control over macrophage lifespan, preventing potential adverse effects. Our three‐step genetic reprogramming strategy has thus established a myeloid progenitor‐based CAR‐macrophage platform that is characterized by its ease of genetic manipulation, rapid large‐scale production, abbreviated preparation cycle, stable uniformity, and favorable safety profile. This platform may provide significant contributions to the development of myeloid progenitor‐derived cancer immunotherapies.

## 2 Results

### 2.1 Scalable Production of Anti‐CEA‐CAR Macrophages using Genetically Engineered Bone Marrow Progenitors

**Figure 1:**
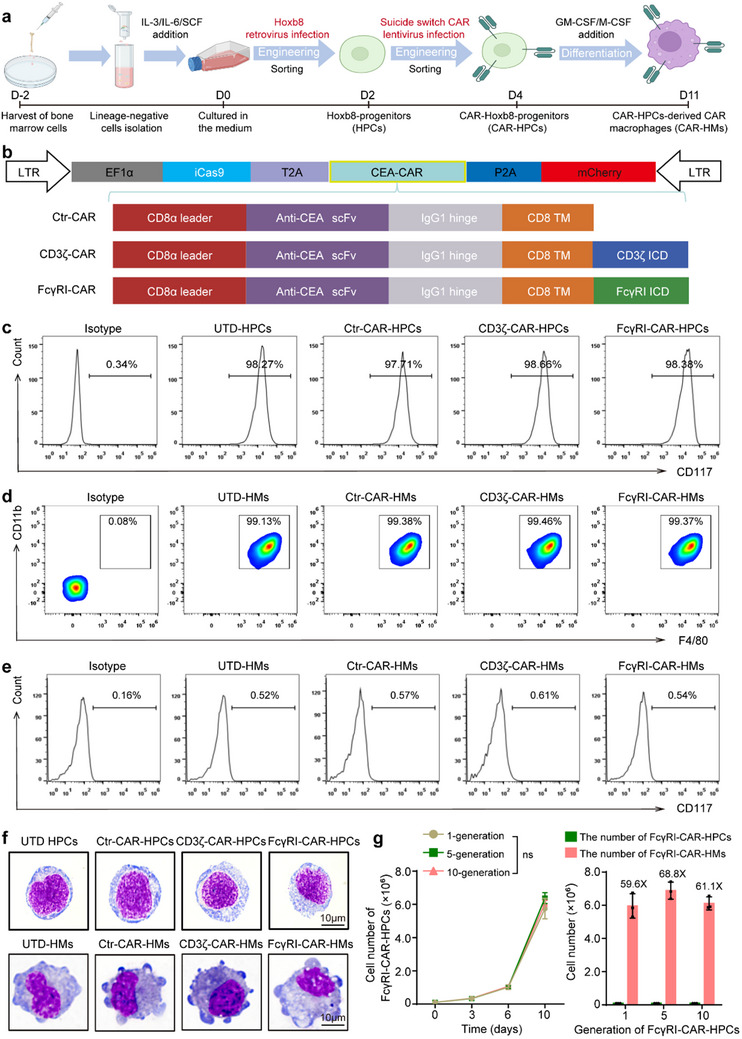
Efficient generation of suicide‐switch anti‐CEA‐CAR macrophages from CAR‐HPCs. a) Schematic diagram of CAR‐HPCs construction and differentiation into CAR‐HMs. b) Lentiviral vector constructs for iCas9‐Anti‐CEA‐CAR expression in HPCs. c) Flow cytometry analysis of the stemness marker CD117 in CAR‐HPCs. UTD, untreated. d,e) Flow cytometry analysis of macrophage markers (F4/80 and CD11b) and stemness marker (CD117) in CAR‐HMs derived from CAR‐HPCs. f) Morphology of CAR‐HPCs and CAR‐HMs was characterized by Wright‐Giemsa staining. Scale bar, 10 µm. Data in panels (c‐f) are from 3 independent biological replicates. g) Proliferation of CAR‐HPCs across generations (1st, 5th, and 10th) was examined by flow cytometry (left). FcγRI‐CAR‐HPCs (1×10^5^) from each generation were cultured and expanded for 10 days before differentiation into FcγRI‐CAR‐HMs. The yield of FcγRI‐CAR‐HMs was quantified using flow cytometry (right). Data in panel (g) are from three independent samples. Data are shown as mean ± standard deviation (SD). Statistical analyses were performed using two‐way ANOVA test with Sidak's multiple comparisons test for panel (g, left). ns, not significant.

**Figure** **1**a,b shows the schematic illustration of the process for generating suicide switch‐equipped anti‐CEA‐CAR macrophages (CAR‐HMs) derived from myeloid progenitors. Initially, lineage‐negative progenitors were isolated from mouse bone marrow cells using magnetic bead sorting. The cells were first infected with a retrovirus encoding an estrogen receptor‐fused Hoxb8. Its activity was induced by 4‐hydroxytamoxifen (4‐OHT), leading to the generation of self‐renewing myeloid progenitors that maintained stemness and expressed CD117. The single‐cell clone of HPCs was obtained by fluorescence activated cell sorting (FACS) (Figure S1a–e, Supporting Information). After the addition of GM‐CSF/M‐CSF, these HPCs differentiated into macrophages, excluding other dendritic cell (DC) or neutrophil lineages (Figure S1f–i and Table S1, Supporting Information).

Next, lentiviral vectors encoding macrophage‐specific CARs targeting CEA‐featuring either CD3ζ or FcγRI signaling domains and the suicide gene iCas9 were transduced into Hoxb8‐engineered myeloid progenitors. The previously described FACS and expansion steps were repeated, yielding CAR‐expressing progenitors with transduction efficiencies exceeding 99%, including CD3ζ‐CAR‐HPCs and FcγRI‐CAR‐HPCs (Figure S2a,b, Supporting Information). This process did not affect the stemness and proliferation of the HPCs (Figure 1c; Table S2, Supporting Information). A truncated CAR lacking the intracellular domain was used as a negative control. CAR‐HPCs were then induced to differentiate into CAR‐macrophages (CD3ζ‐CAR‐HMs or FcγRI‐CAR‐HMs) by adding GM‐CSF and M‐CSF. The resulting cells exhibited a high proportion of CD11b⁺F4/80⁺ populations, minimal CD117 expression, and typical macrophage morphology (Figure 1d–f). These CAR‐HPCs successfully differentiated into macrophages and remained viable for at least 10 days (Figure S2c,d, Supporting Information). The expression of Hoxb8 and the proliferative capacity of CAR‐HPCs‐derived macrophages did not recover following reintroduction of 4‐OHT, suggesting their suitability for safety in subsequent in vivo applications (Figure S2e,f, Supporting Information). The proliferation rates of FcγRI‐CAR‐HPCs were consistent across different generations (1, 5, and 10). After a 10‐day expansion, CAR‐HMs increased ≈ 60‐fold compared to the initial CAR‐HPCs (Figure 1g). Furthermore, CAR‐HPCs cryopreserved for over six months maintained high viability (over 85%), stemness (CD117), and CAR expression upon thawing (Figure S2g,h, Supporting Information). Upon revival, CAR‐HPCs also efficiently differentiated into macrophages (Figure S2i, Supporting Information). These results demonstrate that our three‐step strategy enables the rapid and large‐scale generation of CAR‐macrophages from myeloid progenitors.

### 2.2 Engineered Hoxb8‐CAR‐Macrophages Exhibited Robust Anti‐Tumor Activity In Vitro

**Figure 2:**
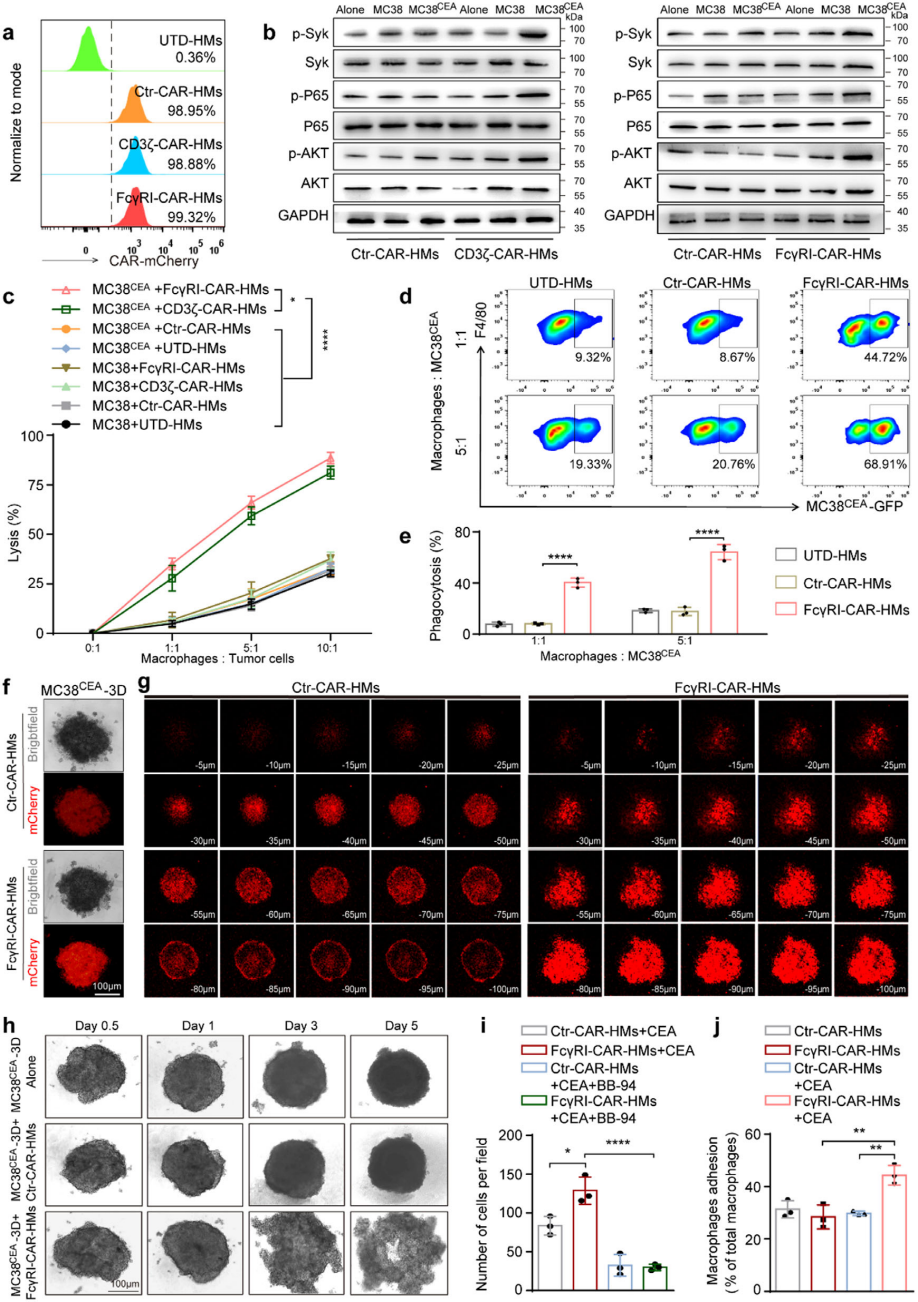
In vitro assessment of anti‐tumor capability of FcγRI‐CAR‐HMs. a) Flow cytometry analysis of CAR (mCherry) expression in CAR‐HMs. b) CAR signal activation in FcγRI‐CAR‐HMs and CD3ζ‐CAR‐HMs after the co‐culture with MC38^CEA^ cells (Macrophages:Tumor cell ratio = 5:1) for 48 h. c) Different types of CAR‐HMs were co‐cultured with MC38^CEA‐Luci^ cells at different ratios for 24 h. Tumor cell lysis was calculated by examining bioluminescence intensity. d,e) Flow cytometry analysis of FcγRI‐CAR‐HMs‐mediated phagocytosis of MC38^CEA^ tumor cells after 24 h co‐culture at the indicated ratios. f,g) CAR‐HMs (2 × 10^5^/well) were added to 24‐well plates containing 3D MC38^CEA^ spheroids. Z‐scan confocal imaging technique was utilized to examine CAR‐HMs (Red) adherence and infiltration into 3D tumor spheroids after 48 h. Scale bar, 100 µm. h) The microscope observation of 3D MC38^CEA^ spheroids co‐cultured with CAR‐HMs over time. Scale bar, 100 µm. i) CEA‐activated FcγRI‐CAR‐HMs or Ctr‐CAR‐HMs (5 × 10^4^ cells) were seeded into the upper chambers, which had been pre‐coated with gelatin (0.1%) and lined with a confluent monolayer of HUVECs. In some chambers, Batimastat (BB‐94, 10 nм) was added to the upper compartment. After 12 h, migrated FcγRI‐CAR‐HMs were fixed, stained with crystal violet, and counted. j) HUVECs (1 × 10^6^ cells well^−1^) were seeded into 6‐well plates and cultured until ≈ 90% confluent. Then, CEA‐activated FcγRI‐CAR‐HMs or Ctr‐CAR‐HMs (2 × 10^5^) were added to HUVEC‐formed membranes for 1 hour. Adhered macrophages were counted to evaluate adhesion capability. *n* = 3 biologically independent samples for panels (a, c–j). Data are shown as means ± SD. Statistical analyses were performed using one‐way ANOVA test with Tukey's multiple comparisons test for panels (e,i,j) and one‐way ANOVA test with Dunnett's multiple comparisons test for panel (c). Significance: ^*^ *P* < 0.05, ^**^ *P* < 0.01, ^****^ *P* < 0.0001.

To evaluate CEA‐dependent CAR‐HMs activation, colorectal cancer cell lines (MC38^CEA^ and CT26^CEA^) that stably express high levels of CEA were generated following published protocols (Figure S3, Supporting Information).^[^ ^36^ ^]^ CARs were stably integrated into CAR‐HMs and CAR‐mediated downstream signaling pathways were significantly activated upon exposure to MC38^CEA^ and CT26^CEA^ cells (**Figure** **2**a,b; Figure S4a, Supporting Information). Notably, FcγRI‐CAR‐HMs activated by CEA showed superior tumor cell lysis capabilities compared to CD3ζ‐CAR‐HMs (Figure 2c; Figure S4b, Supporting Information). This enhanced activity may be due to the FcγRI's role as a key signal for macrophage antibody‐dependent cellular phagocytosis (ADCP), which is more potent in activating macrophages. Therefore, we concentrated on the anti‐tumor effects and mechanisms of FcγRI‐CAR‐HMs in the following studies. Compared to Ctr‐CAR‐HMs and untreated HMs (UTD‐HMs), FcγRI‐CAR‐HMs exhibited enhanced phagocytosis of MC38^CEA^ cells and induced apoptosis in these cells (Figure 2d,e; Figure S4c–e, Supporting Information). The lytic efficiency of FcγRI‐CAR‐HMs against human colorectal cancer cell lines was positively correlated with the levels of CEA expression on these cells (Figure S4f–h, Supporting Information). To simulate the in vivo environment, we constructed a 3D tumor model and studied the adhesion and infiltration abilities of FcγRI‐CAR‐HMs. FcγRI‐CAR‐HMs exhibited significantly higher adhesion and infiltration capacity compared to truncated CAR‐HMs (Figure 2f,g). Our data demonstrate that FcγRI‐CAR‐HMs trigger significant structural disintegration of tumor spheroids after 5 days of co‐culture (Figure 2h). To further validate tumor cell apoptosis, we performed western blot analyses showing upregulated cleaved caspase‐3 and BAX expression with concurrent BCL‐2 downregulation in treated spheroids (Figure S5, Supporting Information). Since intravenously injected CAR‐HMs must first adhere and transmigrate across the vascular endothelium before entering into tumor tissues, we assessed the trans‐endothelial migration and vascular adhesion capacity of CEA‐activated CAR‐HMs. CEA antigen stimulation effectively enhanced the trans‐endothelial migration and vascular adhesion abilities of FcγRI‐CAR‐HMs (Figure 2i,j; Figure S6, Supporting Information). Given the critical role of MMPs activity in leukocyte migration, we also applied BB‐94, a broad‐spectrum MMP inhibitor, in the trans‐endothelial migration assays. Treatment with BB‐94 markedly reduced the transmigration capacity of FcγRI‐CAR‐HMs (Figure 2i).

### 2.3 CEA‐Induced FcγRI‐CAR‐HMs Display a Prominent M1 Phenotype and Upregulate Immune Activation Genes

**Figure 3:**
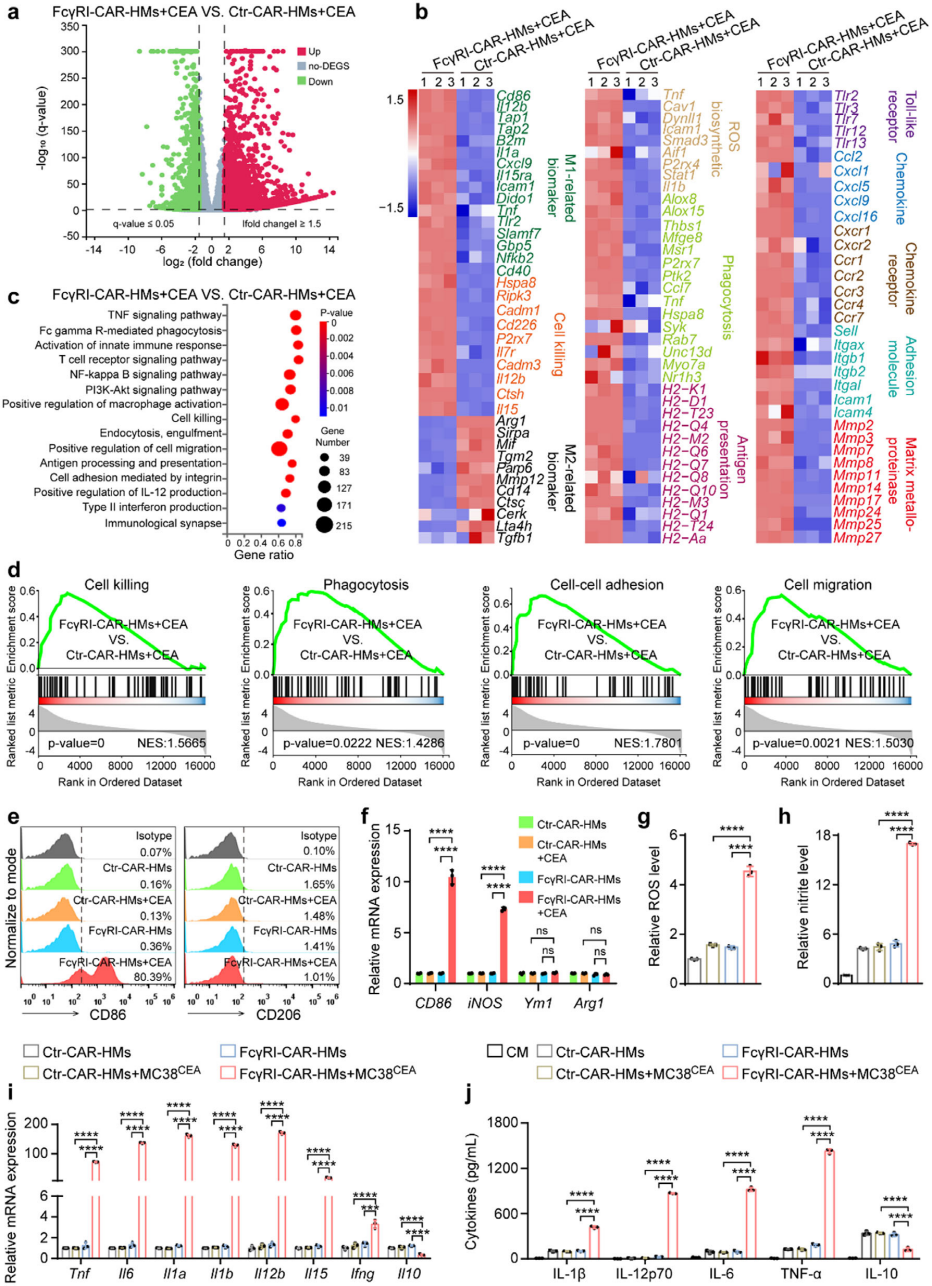
Transcriptional and functional characterization of CAR‐HMs after CEA stimulation. a) Volcano plots showing comparison of the global gene transcriptional profiling of FcγRI‐CAR‐HMs and Ctr‐CAR‐HMs treated with CEA (100 ng mL^−1^) for 48 h. b) Heatmaps of differentially expressed immune‐related genes (DEGs) in FcγRI‐CAR‐HMs and Ctr‐CAR‐HMs. c) Gene ontology (GO) terms enriched among DEGs related to immune activation. d) GSEA analysis demonstrating upregulation of genes related to cell killing, phagocytosis, cell‐cell adhesion, and migration. *n* = 3 biologically independent samples. e,f) Flow cytometry and qRT‐PCR analysis of macrophage markers (CD86, CD206, iNOS, Ym1, and Arg1) in FcγRI‐CAR‐HMs and Ctr‐CAR‐HMs treated with CEA (100 ng mL^−1^) for 48 h. g,h) Detection of total ROS production and nitrite levels from macrophages co‐cultured with MC38^CEA^ cells (1:1 ratio) for 48 h using H2DCFDA probe and Griess Reagent System kit. i) qRT‐PCR measurement of mRNA levels in CAR‐HMs stimulated with MC38^CEA^‐conditioned medium for 48 h. j) ELISA determination of cytokine levels in CAR‐HMs co‐cultured with MC38^CEA^ cells (1:1 ratio) for 48 h. *n* = 3 biologically independent samples. CM, conditioned medium. Data are shown as means ± SD. Statistical analysis was performed using one‐way ANOVA test with Tukey's multiple comparisons test for panels (f–j) except panel (f, *Arg1*) and Kruskal‐Wallis test with Dunn's multiple comparisons test for panel (f, *Arg1*). Significance: ^***^ *P* < 0.001, ^****^ *P* < 0.0001, ns, not significant.

The CEA antigen was found to induce morphology changes in FcγRI‐CAR‐HMs through their activation (Figure S7a, Supporting Information). To elucidate the molecular mechanisms of FcγRI‐CAR‐HMs' anti‐tumor activity, we performed transcriptome sequencing on these cells following CEA treatment (**Figure** **3**a). Compared to Ctr‐CAR‐HMs, transcriptional profiling showed that FcγRI‐CAR‐HMs after CEA treatment exhibited robust upregulation of M1‐related markers, genes associated with immune activation and phagocytosis, alongside diminished expression of M2 phenotype‐related genes (Figure 3b,c). Gene Set Enrichment Analysis (GSEA) correlated these findings, highlighting a significant positive correlation in pathways related to anti‐tumor activity, including cell killing, adhesion, migration, and phagocytosis, between FcγRI‐CAR‐HMs and Ctr‐CAR‐HMs (Figure 3d; Figure S7b, Supporting Information). Flow cytometry and qRT‐PCR analyses confirmed that FcγRI‐CAR‐HMs displayed an immune‐activated M1 phenotype and markedly upregulate the expression of chemoattractant, chemokine receptors (such as CCR2 and CCR7) and adhesion molecules (such as LFA‐1 and Integrin β1) (Figure 3e,f; Figure S8a–c, Supporting Information), corroborating transcriptome sequencing results. This may explain that FcγRI‐CAR‐HMs are able to efficiently infiltrate 3D tumor spheroids and exhibit enhanced adhesion and trans‐endothelial migration capabilities. Interestingly, under CEA treatment, FcγRI‐CAR‐HMs showed elevated levels of chemokines compared to Ctr‐CAR‐HMs, implying their enhanced capability in recruiting T cells (Figure S8a, Supporting Information). FcγRI‐CAR‐HMs co‐cultured with MC38^CEA^ cells produced large amounts of reactive oxygen species (ROS) (Figure 3g), NO (Figure 3h), and released significant quantities of immunostimulatory factors, including IL‐1β, IL‐12, IL‐6, and TNF‐α, and decreased IL‐10 level (Figure 3i,j).

### 2.4 FcγRI‐CAR‐HMs Efficiently Infiltrated into the Tumor Tissues

**Figure 4:**
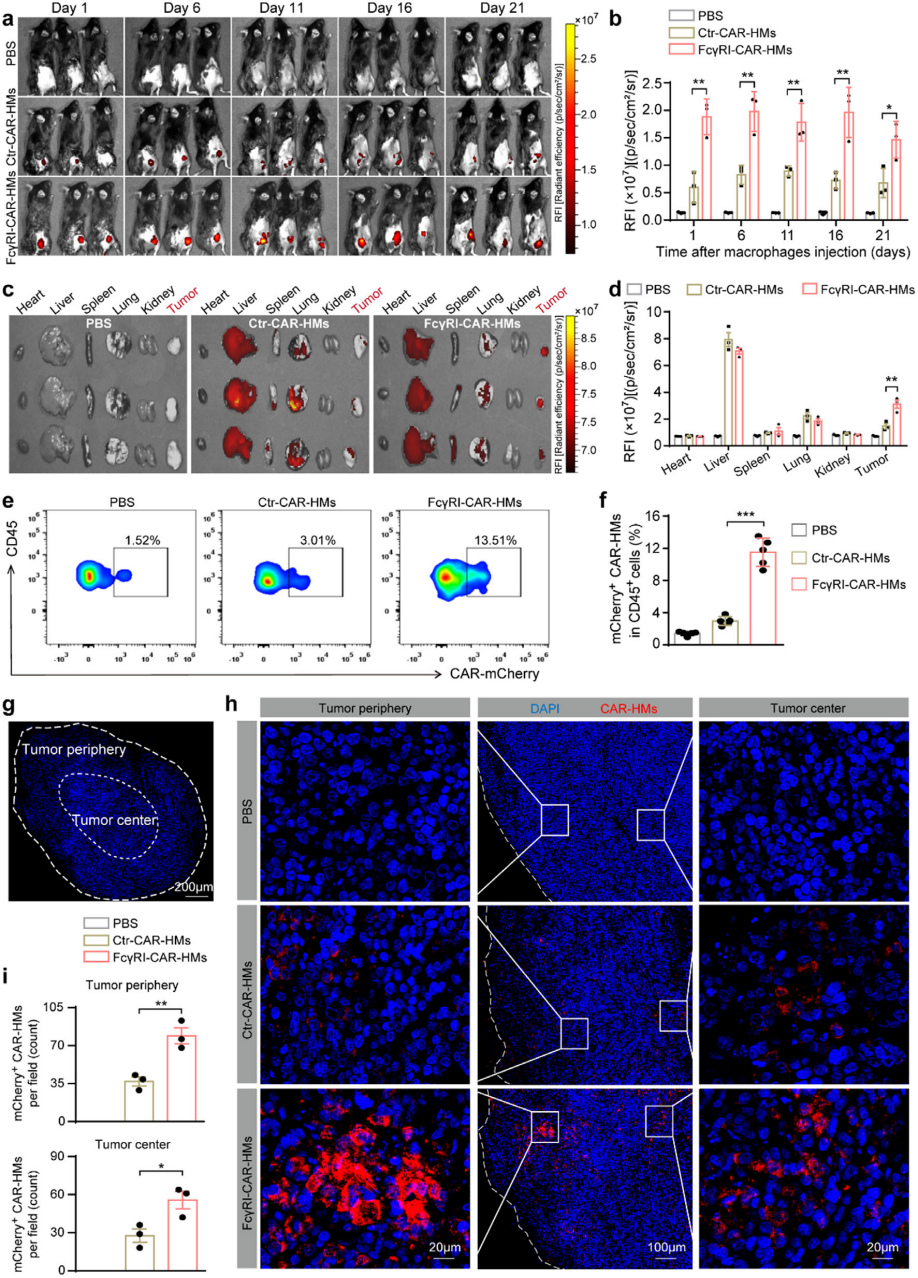
In vivo evaluation of biodistribution and tumor penetrating capability of FcγRI‐CAR‐HMs. a,b) Representative fluorescence images of the MC38^CEA^ tumor bearing mice and quantitative analysis of tumor site fluorescence at the indicated time points after intravenous administration of different DiR‐labelled CAR‐HMs (*n* = 3 mice per group, dose: 1 × 10^7^ cells per mouse). c,d) Representative fluorescence images of the organs and tumor tissues from mice on day 21 post the administration of different CAR‐HMs (*n* = 3 mice per group). e,f) Flow cytometry was used to analyze the ratio of mCherry^+^ CAR‐HMs in CD45^+^ tumor‐infiltrating leukocytes from mice on day 21 after CAR‐HMs administration (*n* = 5 mice per group, dose: 1 × 10^7^ cells per mouse). g,h) Representative fluorescence images of tumor sections from mice on day 21 post injection of FcγRI‐CAR‐HMs and Ctr‐CAR‐HMs. i) Quantitative analysis of count CAR‐HMs at the tumor periphery (distance from tumor edge < 300 µm) and center (distance from tumor edge > 300 µm). Scale bars are included in panels (g–h) for reference. Data are shown as means ± SD. Statistical analysis was performed using one‐way ANOVA test with Tukey's multiple comparisons test for panels (b, d, i) and Brown‐Forsythe and Welch ANOVA tests with Tamhane's T2 multiple comparisons test for panels (f). Significance: ^*^ *P* < 0.05, ^**^ *P* < 0.01, ^***^ *P* < 0.001.

To evaluate the tumor‐homing and infiltration properties of FcγRI‐CAR‐HMs, DIR‐labeled FcγRI‐CAR‐HMs were intravenously injected into MC38^CEA^ tumor‐bearing mice, and fluorescence images were captured at indicated time points. Results in **Figure** **4**a,b showed that FcγRI‐CAR‐HMs exhibited a significantly increased accumulation in tumors compared to Ctr‐CAR‐HMs. Long‐term fluorescence images of mouse organs confirmed that FcγRI‐CAR‐HMs were still detectable in the tumor 21 days post‐administration (Figure 4c,d). Flow cytometry analysis provided further validation of the in vivo imaging data, demonstrating that 13.51% of tumor‐infiltrating leukocytes were mCherry^+^ FcγRI‐CAR‐HMs (Figure 4e,f). Consistent with the fluorescence images of tumor tissues, confocal microscopy examination of frozen sections from MC38^CEA^ tumors revealed that numerous FcγRI‐CAR‐HMs were localized within the tumor tissues and had effectively infiltrated the tumor core (Figure 4g–i). These results further indicate the potent infiltration capability of FcγRI‐CAR‐HMs, highlighting their ability to efficiently enter and persist in TME.

### 2.5 FcγRI‐CAR‐HMs Potently Inhibited Tumor Growth in Colorectal Cancer Model

**Figure 5:**
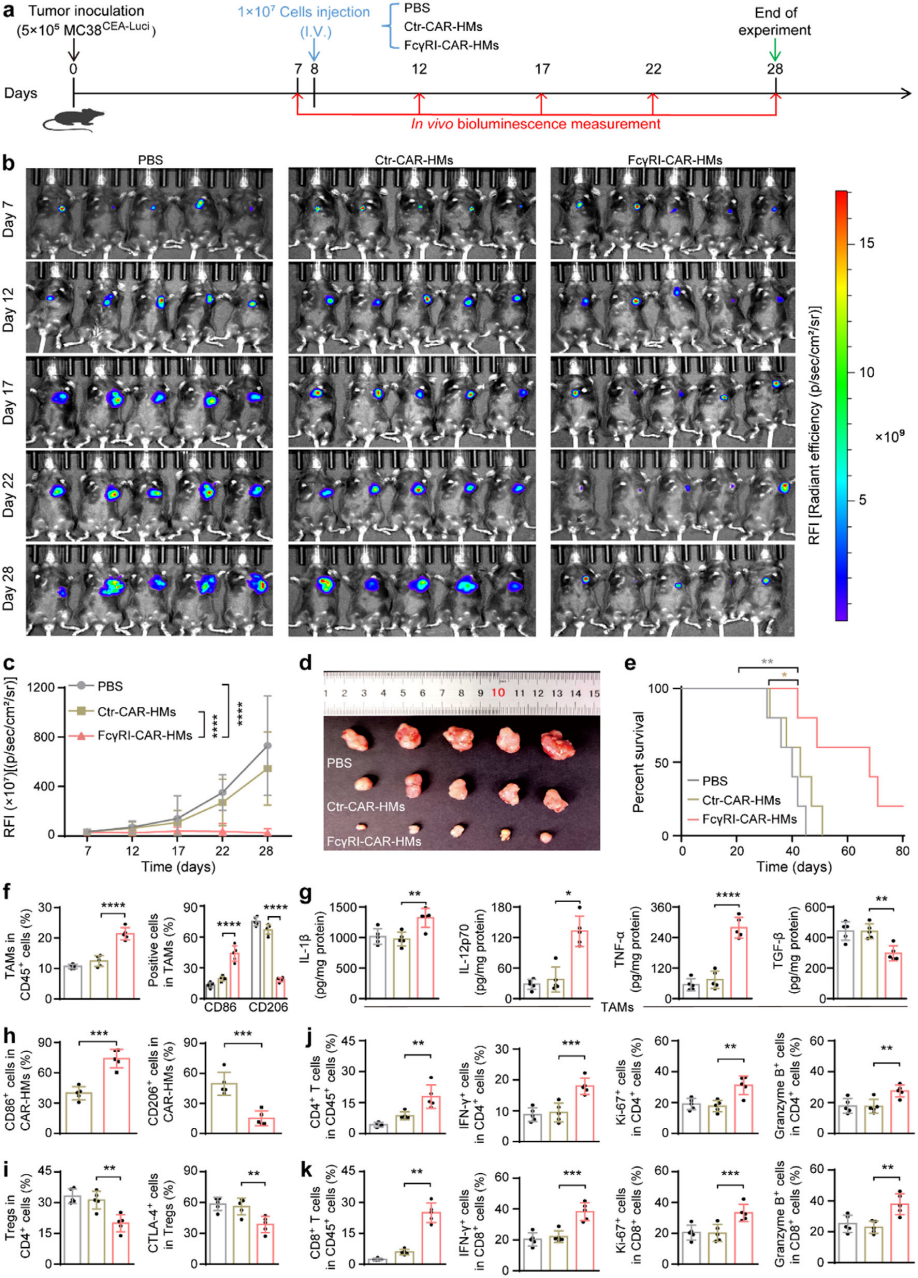
Intravenous injection of FcγRI‐CAR‐HMs effectively suppressed tumor growth and elicited anti‐tumor immune responses in MC38^CEA^ tumor bearing mice. a) Schematic diagram of the experimental design for intravenous injection of various CAR‐HMs (1 × 10^7^ cells per mouse) into MC38^CEA‐Luci^ tumor‐bearing mice on day 8 post tumor cell inoculation. b,c) Bioluminescence imaging and quantification of fluorescence intensity in mice with above‐mentioned treatments. d) Representative images of tumors from mice treated with different CAR‐HMs on day 28 post tumor cell inoculation. e) Survival curves for a separate cohort of mice with the treatment of FcγRI‐CAR‐HMs or Ctr‐CAR‐HMs. Survival data were analyzed by using the log‐rank (Mantel‐Cox) test. f,g) The effects of FcγRI‐CAR‐HMs treatment on the TAMs ratio, phenotype (CD86 and CD206 expression), and cytokines secretion (IL‐1β, IL‐12p70, TNF‐α, and TGF‐β) were evaluated in MC38^CEA^ tumor‐bearing mice on day 28 after tumor model establishment. h) The phenotype of intratumoral CAR‐HMs was assessed by flow cytometry on day 28 following tumor cell inoculation. i–k) The influence of FcγRI‐CAR‐HMs on intratumoral Tregs (proportion of Tregs in CD4^+^ T cells and CTLA‐4 expression within Tregs) and T cell activation and proliferation (proportions of IFN‐γ^+^, Ki‐67^+^, and Granzyme B^+^ cells in CD4^+^/CD8^+^ T cell subsets) were analyzed by flow cytometry on day 28 after tumor model establishment. *n* = 5 mice per group. Data are shown as means ± SD. Statistical analysis was performed using two‐way ANOVA test with Sidak's multiple comparisons test for panel (c), one‐way ANOVA test with Tukey's multiple comparisons test for panels (f, g, i, j, k) except panel (g, IL‐12p70; k, CD8^+^ T cells ratio), Kruskal‐Wallis test with Dunn's multiple comparisons test for panel (g, IL‐12p70), two‐tailed unpaired t test for panel (h), and Brown‐Forsythe and Welch ANOVA tests with Tamhane's T2 multiple comparisons test for panel (k, CD8^+^ T cells ratio). Significance: ^*^ *P* < 0.05, ^**^ *P* < 0.01, ^***^ *P* < 0.001, ^****^ *P* < 0.0001.

The therapeutic efficacy of FcγRI‐CAR‐HMs was initially evaluated in a MC38^CEA^ tumor‐bearing mouse model (**Figure** **5**a). Intravenous injection of FcγRI‐CAR‐HMs effectively inhibited tumor growth and improved survival rates (Figure 5b–e; Figure S9a–c, Supporting Information). Histological examination of tumor sections using H&E and immunohistochemical staining revealed that treatment with FcγRI‐CAR‐HMs led to substantial tumor necrosis and massive tumor cell apoptosis (Figure S9d, Supporting Information). Furthermore, treatment with FcγRI‐CAR‐HMs did not result in any apparent side effects. H&E staining of organs revealed no significant pathological changes. Additionally, there were no notable alterations in body weight, organ index, blood biochemical parameters and serum immunostimulatory cytokine levels (Figure S10, Supporting Information). These findings collectively suggest that FcγRI‐CAR‐HMs are well tolerated in vivo.

To further investigate the impact of FcγRI‐CAR‐HMs on the immune landscape of MC38 tumors, we conducted flow cytometry and ELISA analyses. FcγRI‐CAR‐HMs treatment shifted tumor‐associated macrophages (TAMs) from an immunosuppressive to immunostimulatory phenotype, evidenced by upregulated CD86/IL‐1β/IL‐12/TNF‐α and downregulated CD206/TGF‐β (Figure 5f,g; Figures S11a,b and S12, Supporting Information). Notably, FcγRI‐CAR‐HMs within the tumor exhibited a CD86^high^CD206^low^ phenotype, suggesting that CEA‐mediated FcγRI signaling effectively maintains the pro‐inflammatory M1 phenotype of CAR‐macrophages, even under the immunosuppressive pressures of the TME (Figure 5h; Figure S11d, Supporting Information). In addition, FcγRI‐CAR‐HMs treatment reduced the populations of immunosuppressive cells, including regulatory T cells (Tregs) and myeloid‐derived suppressor cells (MDSCs), and their inhibitory molecules (CTLA‐4, Arg1; Figure 5i; Figure S11e, Supporting Information). FcγRI‐CAR‐HMs treatment also increased the proportions of NK cells, dendritic cells (DCs), and both CD4⁺ and CD8⁺ T cells, accompanied by elevated expression of activation and proliferation markers. These included CD80 and CD86 in DCs, and IFN‐γ, Ki‐67, and Granzyme B in T cells (Figure 5j,k; Figure S11c,f–i, Supporting Information). ELISA results further confirmed significant increases in immunostimulatory cytokines (IL‐12, TNF‐α, IFN‐γ, IL‐1β, IL‐6, and FLT3LG) and the chemokine CXCL9, along with a decrease in TGF‐β level (Figure S11j, Supporting Information). Furthermore, FcγRI‐CAR‐HMs treatment inhibited tumor growth in the HT29 tumor model (Figure S13, Supporting Information). These results indicate that FcγRI‐CAR‐HMs can effectively reshape the TME and exert potent anti‐tumor effects.

### 2.6 FcγRI‐CAR‐HMs Enhanced the Therapeutic Effects of Immune Checkpoint Inhibitors

**Figure 6:**
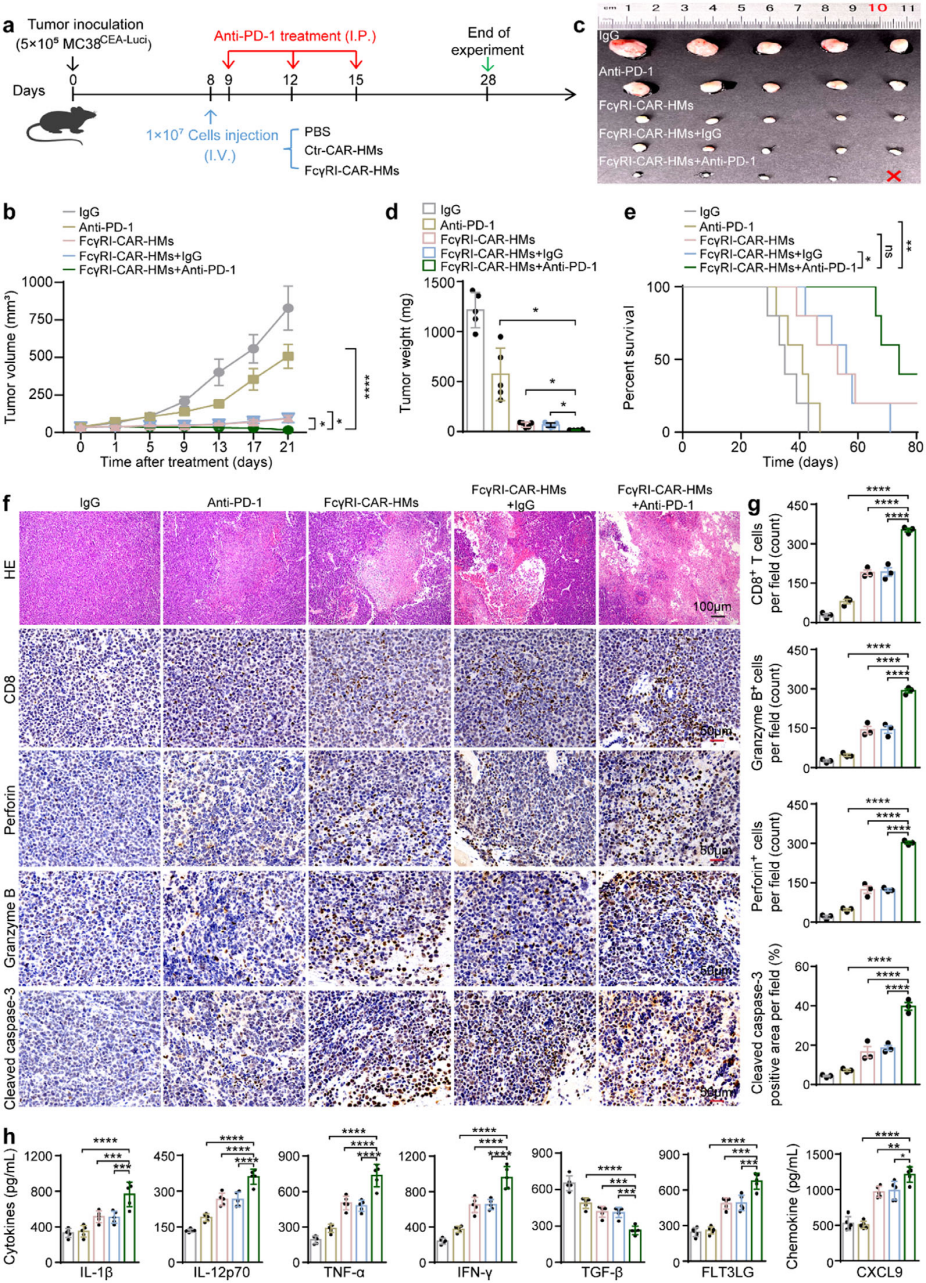
Synergistic anti‐tumor effects of FcγRI‐CAR‐HMs and anti‐PD‐1 antibody therapy in MC38^CEA^ tumor‐bearing mice. a) Schematic diagram of the experimental design for intravenous injection of various CAR‐HMs (1 × 10^7^ cells per mouse) and intraperitoneally injected with anti‐PD‐1 antibody in MC38^CEA^ tumor‐bearing mice. b) Tumor growth curves of mice with above mentioned treatments (*n* = 5 mice per group). c,d) Representative tumor images and corresponding tumor weights from mice with different treatments on day 28 post tumor cell inoculation (*n* = 5 mice per group). e) Survival curves from a separate cohort of mice co‐treated with FcγRI‐CAR‐HMs and anti‐PD‐1 antibody (*n* = 5 mice per group). Survival data were analyzed by using the log‐rank (Mantel‐Cox) test. f,g) Representative H&E and immunohistochemical staining of cytotoxic T cell markers (CD8, Perforin, Granzyme B) and apoptosis‐related marker (Cleaved caspase‐3) in MC38^CEA^ tumor tissues from mice with above mentioned treatments (*n* = 3 mice per group). h) Cytokine and chemokine levels in MC38^CEA^ tumors of mice treated with CAR‐HMs and anti‐PD‐1 antibody were determined by ELISA on day 28 post tumor cell inoculation (*n* = 5 mice per group). Scale bars are included in panel (f) for reference. Data are shown as means ± SD. Statistical analysis was performed using two‐way ANOVA test with Sidak's multiple comparisons test for panel (b), Brown‐Forsythe and Welch ANOVA tests with Tamhane's T2 multiple comparisons test for panel (d), one‐way ANOVA test with Tukey's multiple comparisons test for panels (g, h). Significance: ^*^ *P* < 0.05, ^**^ *P* < 0.01, ^***^ *P* < 0.001, ^****^ *P* < 0.0001, ns, not significant.

As treatment with FcγRI‐CAR‐HMs could effectively reverse the immunosuppressive TME, PD‐1 inhibitors and FcγRI‐CAR‐HMs were co‐administered for the treatment of the MC38^CEA^ tumor model (**Figure** **6**a). The combination treatment exhibited superior therapeutic effects in suppressing tumor growth and improving survival rates compared to either PD‐1 inhibitor or FcγRI‐CAR‐HMs monotherapy (Figure 6b–e). The combination treatment further enhanced tumor necrosis and tumor cell apoptosis (Figure 6f,g). To elucidate the mechanisms underlying the synergistic effects of FcγRI‐CAR‐HMs and PD‐1 inhibitors, we conducted additional analyses. The combined therapy significantly elevated the expression of immunostimulatory cytokines, such as IL‐12 and TNF‐α, in TAMs (Figure S14a, Supporting Information). Moreover, co‐treatment further promoted the infiltration, activation, and proliferation of CD8⁺ T cells, while reducing the proportions of immunosuppressive cells, including MDSCs and Tregs (Figure 6f,g; Figure S14b–g, Supporting Information). Consistent with these findings, levels of immunostimulatory cytokines and chemokines in tumor tissues were markedly increased following combination therapy (Figure 6h). Therefore, the addition of PD‐1 inhibitors blocked an alternative pathway of tumor‐mediated immune evasion, suggesting that the integration of FcγRI‐CAR‐HMs with PD‐1 blockade holds significant promise for enhancing cancer immunotherapy outcomes.

### 2.7 The Evaluation of iCas9 Safety Switch in FcγRI‐CAR‐HMs

**Figure 7:**
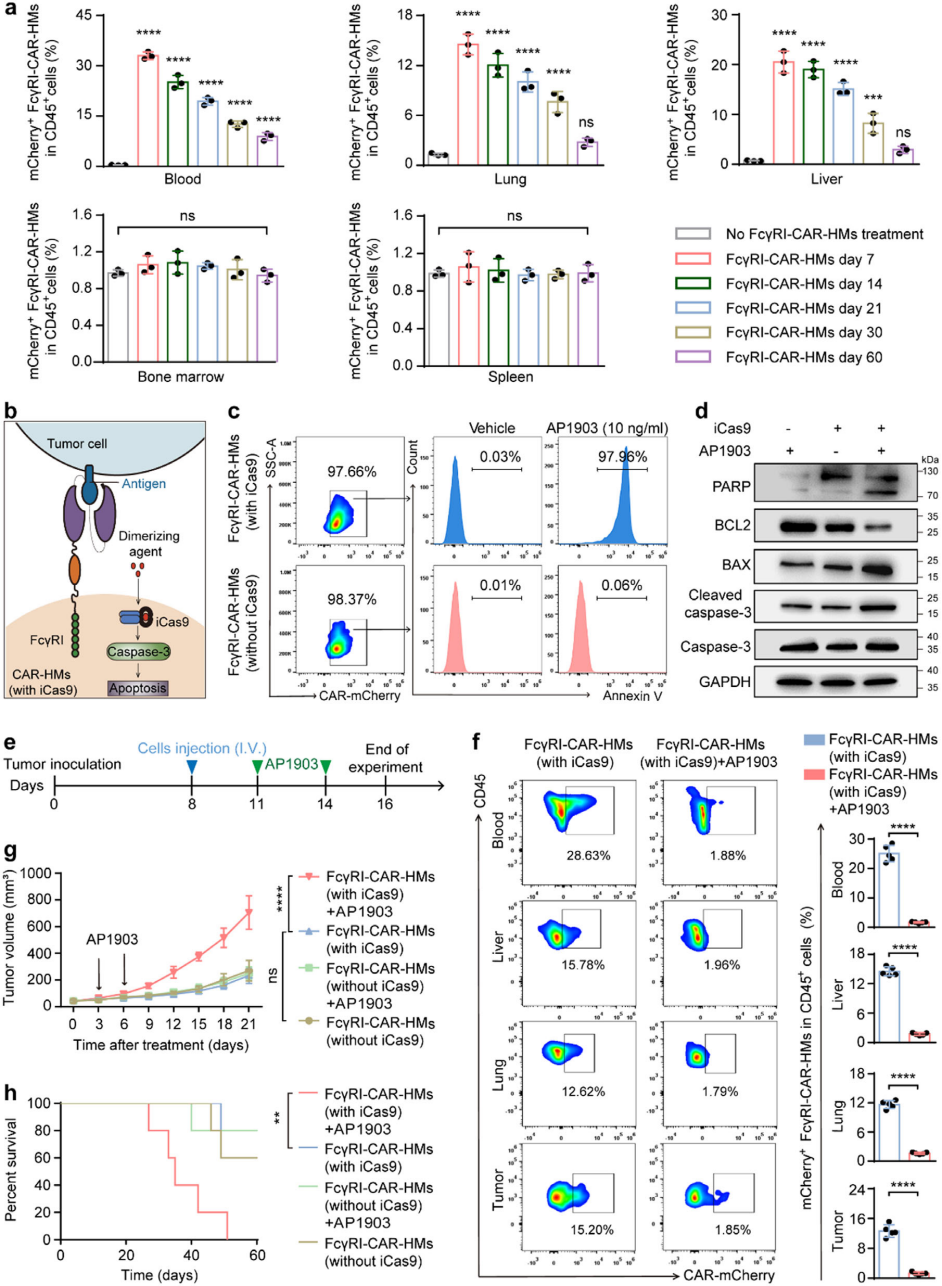
AP1903 effectively mediated the clearance of FcγRI‐CAR‐HMs. a) Flow cytometry was used to analyze the ratio of FcγRI‐CAR‐HMs in CD45^+^ leukocytes from the blood and organs (lung, liver, bone marrow and spleen) of mice at indicated time points after CAR‐HMs administration (1 × 10^7^ cells per mouse). *n* = 3 mice per group. Comparisons were made between FcγRI‐CAR‐HMs treatment at various time points and a no‐treatment control group. b–d) Flow cytometry and western blotting analysis of apoptotic ratios and related apoptotic proteins in FcγRI‐CAR‐HMs with or without iCas9 after a 6 h exposure to AP1903 (10 ng mL^−1^). *n* = 3 biologically independent samples. e) Schematic diagram depicting the experimental timeline: FcγRI‐CAR‐HMs (1 × 10^7^ cells per mouse) were injected intravenously into MC38^CEA^ tumor‐bearing mice on day 8 post tumor cell inoculation, followed by intraperitoneal injection of AP1903 (5 mg kg^−1^) on days 11 and 14. f) Flow cytometry was used to analyze the ratio of FcγRI‐CAR‐HMs in CD45^+^ leukocytes from the tumor and organs (lung, liver, and blood) of mice with above‐mentioned treatments (n = 5 mice per group). g) Tumor growth curves of mice with different treatments (*n* = 5 mice per group). (h) Survival curves for a separate cohort of mice co‐treated with FcγRI‐CAR‐HMs and AP1903 (*n* = 5 mice per group). Survival data were analyzed by using the log‐rank (Mantel‐Cox) test. Data are shown as means ± SD. Statistical analysis was performed using one‐way ANOVA test with Tukey's multiple comparisons test for panel (a), two‐tailed unpaired t test with Welch's correction for panel (f) and two‐way ANOVA test with Sidak's multiple comparisons test for panel (g). Significance: ^**^ *P* < 0.01, ^***^ *P* < 0.001, ^****^ *P* < 0.0001, ns, not significant.

A tissue distribution study revealed that FcγRI‐CAR‐HMs maintained activity in tumor‐bearing mice for up to 21 days. Flow cytometry results detected that FcγRI‐CAR‐HMs persisted in peripheral blood, lung, and liver tissues for up to 60 days (**Figure** **7**a; Figure S15, Supporting Information). This indicates the stable and durable presence of FcγRI‐CAR‐HMs in vivo. However, to prevent potential adverse effects like secondary T‐cell lymphomas and cytokine‐release syndrome (CRS) observed in CAR‐T therapy, precise control over the in vivo survival time of FcγRI‐CAR‐HMs is necessary. Treatment with AP1903 alone induced apoptosis in over 90% of FcγRI‐CAR‐HMs in vitro (Figure 7b–d; Figure S16, Supporting Information). Subsequently, we evaluated the rapid induction of FcγRI‐CAR‐HMs cells apoptosis by AP1903 in vivo (Figure 7e). Under AP1903 treatment, FcγRI‐CAR‐HMs became undetectable in vivo and failed to inhibit tumor growth, leading to decreased survival rates in mice (Figure 7f–h). These results confirm that AP1903 can effectively eliminate FcγRI‐CAR‐HMs both in vitro and in vivo, potentially avoiding severe side effects during treatment with FcγRI‐CAR‐HMs.

## 3 Discussion

Macrophages, with owing to their innate tropism for tumors‐tropic properties and a pivotal role in bridging innate and adaptive immunity, have attracted considerable interest in the treatment of solid tumors.^[^ ^25^, ^37^ ^]^ The FDA has granted approval for two CAR‐macrophage‐related therapies: MCY‐M11, which modifies peripheral blood mononuclear cells including macrophages, and CT‐0508, an autologous anti‐HER2‐CAR macrophage therapy. However, CT‐0508, derived from patient peripheral blood, was discontinued by Carisma Therapeutics due to financial constraints. While specific reasons were not disclosed, the discontinuation likely reflects the inherent challenge in efficiently infiltrating into tumor tissue and obtaining sufficient macrophages for modification, as these terminally differentiated cells lack proliferative capacity, rendering large‐scale production costly and complex.^[^ ^24^ ^]^ Consequently, the company redirected efforts to in vivo CAR‐monocyte modification. Therefore, advancing CAR‐macrophages in clinical applications requires identifying a safe, reliable, and scalable macrophage source that can be stably engineered to express CAR genes and efficiently infiltrate tumors.

In this study, we used CAR‐modified, Hoxb8 conditional bone marrow‐derived myeloid progenitors to generate CAR‐macrophages, thereby expanding the macrophage sources. Hoxb8, a member of the Hox family with a conserved homeodomain motif, inhibits myeloid differentiation and maintains cell survival and proliferation by regulating c‐Myc and Bim.^[^ ^38^, ^39^ ^]^ The combination of tamoxifen‐dependent Hoxb8 activation and SCF effectively sustains the self‐renewal of myeloid progenitors.^[^ ^39^ ^]^ Our research found that Hoxb8 conditional myeloid progenitors did not undergo exhaustion like primary macrophages after CAR gene‐modification. Instead, they maintained a high proliferation rate, achieving a 60‐fold expansion within 10 days, significantly outpacing CAR‐modified iPSCs, which require 20–30 days for a 50‐fold expansion.^[^ ^27^ ^]^ The rapid expansion of CAR‐Hoxb8 myeloid progenitors supports large‐scale CAR‐macrophages production. Moreover, primary macrophages have limited CAR transduction efficacy,^[^ ^40^, ^41^ ^]^ posing a challenge to obtaining high‐purity CAR‐macrophages. In contrast, Hoxb8 conditional myeloid progenitors exhibit high transduction efficacy (over 40%). Importantly, CAR‐Hoxb8 conditional myeloid progenitors maintain a stable phenotype even after multiple generations of expansion. Previous studies have shown that Hoxb8 progenitors retain normal karyotypes and can differentiate into macrophages after 850 generations of expansion further supporting our results.^[^ ^39^, ^42^ ^]^ Selecting a single clone allows for acquiring a genetically homogeneous population.^[^ ^25^, ^43^ ^]^ These quality‐stable CAR‐Hoxb8 progenitors can efficiently differentiate into CAR‐macrophages under specific cytokines (e.g., GM‐CSF and M‐CSF). They express typical macrophage markers. Our study also demonstrated that Hoxb8 progenitors maintain high viability after cryopreservation. Unlike iPSCs‐derived cells, which may face increased heterogeneity and changes in differentiation potential during long‐term culture and expansion,^[^ ^27^ ^]^ CAR‐Hoxb8 progenitors quickly differentiate into CAR‐macrophages after short‐term induction. This significantly reduces preparation time and enhances the efficiency and scope of “off‐the‐shelf” CAR‐macrophage‐based therapies for solid malignancies.

Effective treatment strategies for advanced CRC remain limited.^[^ ^44^ ^]^ Given that CRC cells specifically overexpress CEA, an important marker for early screening and prognosis of CRC,^[^ ^45^ ^]^ we engineered anti‐CEA CAR‐macrophages to target and eliminate CRC cells. Previous clinical trials with anti‐CEA CAR‐T cells have demonstrated safety, affirming the reliability of CEA as a target for adoptive CAR‐cell therapy. However, CRC patients have not seen significant benefits from anti‐CEA CAR‐T cell therapy, as these cells struggle with persistence and infiltration into tumor tissues.^[^ ^46^, ^47^ ^]^ Similarly, clinical studies with another type of CAR‐T cells (anti‐TAG72) showed that CAR‐T cells fail to penetrate the core of large CRC tumors.^[^ ^48^ ^]^ In our in vitro and in vivo experiments, FcγRI‐CAR‐HMs exhibit enhanced adhesion and trans‐endothelial migration capabilities, efficiently infiltrated 3D tumor spheroids and tumor tissues, and maintain long‐term persistence within the tumor. The increased tumor infiltration capacity of FcγRI‐CAR‐HMs might be attributed to the following aspects. Firstly, macrophages derived from myeloid progenitors inherently demonstrate greater infiltration capacity than primary bone marrow‐derived macrophages (BMDMs).^[^ ^42^ ^]^ Secondly, FcγRI‐CAR‐HMs stimulated with CEA expressed higher levels of migration‐related chemokine receptors (e.g., CCR2), tumor matrix‐degrading enzymes (e.g., MMP14) and adhesion molecules (e.g. LFA‐1) compared to HMs infected with an empty retroviral vector.^[^ ^49^, ^50^, ^51^, ^52^, ^53^, ^54^ ^]^ Once recruited by chemokines secreted from the tumor microenvironment, FcγRI‐CAR‐HMs adhere to endothelial cells through interactions between LFA‐1/integrins and ICAMs, and subsequently undergo trans‐endothelial migration and deep tumor infiltration facilitated by MMPs activity. Therefore, FcγRI‐CAR‐HMs offer unique advantages in treating solid tumors, particularly large ones.

Macrophages eliminate tumor cells through multiple mechanisms, including direct phagocytosis, cytotoxic molecule secretion, and T cell activation.^[^ ^17^, ^21^, ^25^ ^]^ Therefore, evaluating the anti‐tumor activity of CAR‐macrophages requires both in vitro and in vivo analyses. For in vitro studies, we employed co‐culture systems of macrophages with tumor cells and 3D tumor spheroids to assess phagocytosis, tumor cell lysis, and apoptosis. FcγRI‐CAR‐HMs efficiently infiltrated spheroids, exhibited robust phagocytosis, and showed potent cytotoxicity. Importantly, they maintained an immune‐activated phenotype when co‐cultured with MC38^CEA^ cells, indicating that CEA activation of FcγRI signaling outweighs immunosuppressive effects of MC38‐secreted factors. The anti‐tumor efficacy of FcγRI‐CAR‐HMs was evaluated in immunodeficient nude mice and immunocompetent C57BL/6J mice bearing subcutaneous tumors. Treatment with FcγRI‐CAR‐HMs led to marked tumor regression and prolonged survival in both models. Notably, FcγRI‐CAR‐HMs retained an M1 phenotype (CD86^hi^CD206^low^) in the immunosuppressive TME and remodeled the TME to promote T cell infiltration and activation, as shown by increased expression of immunostimulatory cytokines (e.g., IL‐12p70 and TNF‐α) and chemokines (e.g., CXCL9).^[^ ^55^, ^56^, ^57^, ^58^, ^59^ ^]^ Taken together, these in vitro and in vivo results demonstrate that FcγRI‐CAR‐HMs exert direct anti‐tumor effects and reshape the immunosuppressive TME to promote adaptive immune responses.

Our preclinical models demonstrate that FcγRI‐CAR‐HMs exert anti‐tumor effects primarily through direct tumor cell killing and activation of T cell‐mediated immune responses. These findings suggest two key directions for future combination therapies. First, co‐administering inhibitors targeting myeloid‐specific immune checkpoints, such as antibodies against CD47, SIGLEC family and LILRB family, can further enhance the cytotoxic capacity of CAR‐macrophages.^[^ ^60^ ^]^ For example, combining iPSC‐derived CAR‐macrophages with anti‐CD47 antibodies has been shown to induce significant tumor regression in ovarian cancer models.^[^ ^61^ ^]^ Second, using immune checkpoint inhibitors targeting the PD‐1/PD‐L1 and CTLA‐4 pathways can block tumor‐mediated immune evasion and enhance T cell‐mediated anti‐tumor immunity. In our study, co‐treatment with FcγRI‐CAR‐HMs and anti‐PD‐1 antibodies significantly promoted T cell activation and proliferation, improving therapeutic efficacy. Similar results were observed in mouse breast cancer models, supporting the potential of this combination.^[^ ^18^ ^]^ Collectively, these findings highlight the promising therapeutic potential of combining CAR‐macrophages with other immunotherapeutic agents.

Safety is a primary concern in adoptive cell therapy. Our studies indicated that CAR‐HMs persisted in tumor tissues for up to 21 days, which is essential for their antitumor activity. However, accumulation was also observed in non‐tumor tissues, such as the liver and lungs, consistent with previous reports.^[^ ^19^, ^24^ ^]^ Given that activated CAR‐HMs may secrete pro‐inflammatory cytokines (e.g., IL‐6 and TNF‐α), their long‐term persistence in normal tissues could potentially cause off‐target effects. However, no significant changes were detected in serum biochemistry, organ histopathology, or peripheral cytokine levels in CAR‐HMs treated mice (Figure S10, Supporting Information). These results align with prior evidence from preclinical models and phase I clinical trials, which have demonstrated a favorable safety profile for CAR‐macrophages.^[^ ^17^, ^18^ ^]^ The observed safety may be attributed to the unique biological characteristics of macrophages, including their limited expansion capacity, intrinsic regulatory functions via the secretion of anti‐inflammatory cytokines like IL‐10, and lack of TCR expression, which reduces the risk of graft‐versus‐host disease (GvHD).^[^ ^62^ ^]^ To further enhance safety, we implemented a dual control strategy: the tamoxifen‐inducible Hoxb8 system to control myeloid progenitors proliferation, and the iCas9 suicide switch to promptly eliminate CAR‐HMs in cases of CRS or neurotoxicity.^[^ ^63^, ^64^, ^65^ ^]^ This strategy provides stringent control over CAR‐HMs persistence and reinforces the safety of this therapeutic platform.

Despite the promising antitumor efficacy of CAR‐HMs in our preclinical models, several challenges must be addressed for successful clinical translation. First, the Hoxb8/tamoxifen system, which controls cell proliferation, needs to be validated in human bone marrow progenitors to ensure precise regulation. Second, developing multi‐antigen‐targeting and logic‐gated CARs can address antigen escape and off‐target effects. Incorporating drug‐inducible circuits into CAR designs could enable dynamic modulation of CAR activity, potentially reducing the need for repeated administrations.^[^ ^66^ ^]^ Third, using CRISPR to delete metabolic checkpoints (e.g., ACOD1) or blocking the SIRPα‐CD47 axis can improve macrophage antitumor activity.^[^ ^61^ ^]^ Finally, a thorough assessment of CAR‐HMs efficacy and safety in humanized mouse models is essential. Addressing these issues will be critical for accelerating the clinical translation of CAR‐HMs.

In conclusion, this study demonstrates that anti‐CEA‐CAR genetically engineered myeloid progenitors enable scalable production of high‐quality, tumoricidal, and safe CAR‐macrophages. Future development of human myeloid progenitors based on this strategy may have great potential as a valuable tool in cancer therapy.

## 4 Experimental Section

### Reagents and Cell Culture

Recombinant mouse IL‐3, IL‐6, M‐CSF, GM‐CSF and SCF were obtained from PeproTech (Rocky Hill, NJ, USA). 4‐OHT (cat. no: H6278), retinoic acid (cat. no.: 302‐79‐4) and beta mercaptoethanol (cat. no.: E2758) were purchased from Sigma–Aldrich (St. Louis, MO, USA). Blasticidin S (cat. no.: 60218ES10) and puromycin (cat. no.: 60209ES10) were purchased from Yeasen Biotechnology (Shanghai, China). AP1903 (rimiducid) was obtained from MedChemExpress (cat. no.: HY‐16046, Monmouth Junction, NJ, USA). All cell culture medium, fetal bovine serum (FBS) and penicillin/streptomycin/glutamine (PSG, 100^*^) were sourced from Thermo Fisher Scientific (Grand Island, CA, USA). Caco2 (RRID: CVCL_0025), SW480 (RRID: CVCL_0546), SW620, MC38, CT26 (RRID: CVCL_7254), HCT116 (RRID: CVCL_0291) and HT29 (RRID: CVCL_0320) were obtained from the Cell Bank of Type Culture Collection of Chinese Academy of Sciences (Shanghai, China). The cells were cultured in the corresponding medium: HCT116 and HT29 in McCOY's 5A medium with FBS (10%), MC38 and CT26 in RPMI 1640 medium with FBS (10%), SW480 and SW620 in Leibovitz's L‐15 medium with FBS (10%), Caco2 in MEM medium with Non‐Essential Amino Acids (NEAA, 1%) and FBS (20%). All human cell lines underwent verification through STR DNA profiling, and PCR assays were conducted to check for mycoplasma contamination.

### Construction of Lentiviral Vector and Stable Colorectal Cell Lines

The murine stem cell virus (MSCV) retroviral vector co‐expressing estrogen receptor‐fused Hoxb8 and GFP and the corresponding empty retroviral vector were generated by NewHelix Biotech (Shanghai, China). Lentivirus vectors encoding luciferase (pSLenti‐EF1α‐Luciferase‐P2A‐BSR) and human CEA (pSLenti‐EF1α‐eGFP‐T2A‐puro vector) were constructed by OBiO Technology (Shanghai, China). The backbone of anti‐CEA‐CAR was constructed followed this pattern from N terminus to C terminus: CD8α signal sequence (amino acids 1–27), CEA‐specific scFv nucleotide sequence, IgG1 hinge region (amino acids 98–110) and CD8 transmembrane region sequence (amino acids 197–217), and the intracellular CD3ζ structural domain (amino acids 52–164) or the FcγRI structural domain (amino acids 321–404). The anti‐CEA antigen CAR plus the suicide gene iCas9 was also constructed according to a previous report.^[^ ^67^ ^]^ CAR lacking the intracellular domain was used as the control CAR. All CARs were synthesized by Aikon Biotech Company (Suzhou, China) and cloned into the Lenti‐EF1a‐P2A‐mCherry vector. MC38 and CT26 cells were infected with lentivirus vectors carrying human CEA. MC38^CEA^ and CT26^CEA^ cell lines were selected in culture medium containing puromycin (1 µg mL^−1^). The expression levels of CEA in MC38^CEA^, CT26^CEA^ cells and human CRC lines were examined by flow cytometry, fluorescent microscope or qRT‐PCR. To detect macrophage‐mediated lysis and in vivo tumor growth, we utilized viral vectors carrying luciferase to infect the specified cells (MC38^CEA^, CT26^CEA^, HT29, HCT116, Caco2, SW620, and SW480). Subsequently, Blasticidin S (1 µg mL^−1^) was employed to select for cell lines stably expressing luciferase.

### Generation of CAR‐Hoxb8‐Progenitors and their Differentiation into CAR‐HMs

Hoxb8 progenitors were constructed as previously described.^[^ ^39^ ^]^ Briefly, bone‐marrow cells from C57BL/6J mice were purified to obtain lineage‐negative progenitors via the Lineage Cell Depletion Kit (cat. no.: 130‐090‐858, Miltenyi Biotec, Koln, Germany). These cells were further cultured in IMDM supplemented with FBS (15%), PSG (1%), IL‐3 (10 ng mL^−1^), IL‐6 (20 ng mL^−1^) and SCF (25 ng mL^−1^) for 48 h. Then, the progenitors were transduced with estrogen receptor‐fused Hoxb8 retrovirus (MOI: 50) for 48 h and cultured with Opti‐MEM medium containing FBS (10%), PSG (1%), SCF (10 ng mL^−1^), GM‐CSF (10 ng mL^−1^), beta mercaptoethanol (30 µм), and 4‐OHT (1 µм). The transduced single cell clone was obtained via Fluorescence Activated Cell Sorting (FACS). Briefly, BD FACSAria^TM^ II flow cytometer was applied to sort 1 cell well^−1^ into 96‐well plates. After sorting and expanding the single cell clones, Hoxb8 progenitors were further infected with CAR lentiviral vectors (MOI: 10) for 48 h. The GFP⁺mCherry⁺ single‐cell clone was generated by repeatedly performing the aforementioned FACS and expansion procedures. The transduction efficiency was analyzed by flow cytometry based on GFP or mCherry fluorescent protein expressed by corresponding vectors. The expression levels of Hoxb8 and CAR in CAR‐HPCs and CAR‐HMs were detected by western blotting and qRT‐PCR, respectively. To obtain differentiated CAR‐HMs, the CAR‐HPCs were washed twice with PBS to remove 4‐OHT and subsequently cultured in RPMI 1640 medium supplemented with M‐CSF (20 ng mL^−1^), GM‐CSF (20 ng mL^−1^), FBS (10%), and PSG (1%). The medium was replaced every two days, and differentiated CAR‐HMs were harvested on the 7th day.

Flow cytometry was used to examine the stemness of HPCs and CAR‐HPCs (CD117), the differentiation efficiency (CD11b and F4/80) and Hoxb8 (GFP) and CAR‐expressing level (mCherry). Diff‐Quick or Wright‐Giemsa staining was applied to HPCs and macrophages with different treatments for the observation of cell morphology. The number of macrophages derived from CAR‐HPCs at different passages was quantified using flow cytometry. The cell viability of CAR‐HMs was examined by a CCK‐8 kit (cat. no.: 40203ES76, Yeasen, Shanghai, China). The cell viability and apoptosis states of FcγRI‐CAR‐HMs after exposure to AP1903 (10 ng mL^−1^) for 6 h were examined by flow cytometry analysis (Annexin V positive), CCK‐8 assay, and western blotting. Detailed information was shown in the Supporting Information.

### The In Vitro Evaluation of CAR‐HMs Mediated Anti‐Tumor Activity

To examine CEA‐mediated activation of CAR‐HMs, CAR‐HMs were treated with CEA (100 ng mL^−1^, cat. no.: CE5‐HF254, ACRO, Beijing, China) for 48 h. CAR‐HMs were harvested for transcriptome sequencing, qRT‐PCR and flow cytometry analysis. Bioinformatic analysis was performed to analysis the phenotypes of CAR‐HMs. CAR‐HMs and luciferase stable‐expressing colorectal cancer cell lines (MC38^CEA^ and CT26^CEA^) were co‐cultured at different effector cells to target cells (Macrophages: Tumor cells) ratios (0:1, 1:1, 5:1, and 10:1) for 24 h (HT29, Caco2, SW620, SW480 and HCT116, Macrophages: Tumor cells ratio = 5:1). ROS and NO produced by CAR‐HMs were examined by the addition of H2DCFDA probe (cat. no.: S0033, Beyotime, Shanghai, China) and using the Griess Reagent System kit (cat. no.: G2930, Promega, Wisconsin, USA) according to the manufacture's instruction. The apoptosis of tumor cells was analyzed using the Annexin V‐APC/7‐AAD fluorescent double staining cell apoptosis detection kit (cat. no.: E‐CK‐A218, Elabscience, Wuhan, China). For the phagocytosis assay, tumor cells and CAR‐HMs were labeled with Cell Tracker CMFDA Dye (cat. no.: C7025, Thermo Fisher Scientific, Grand Island, CA, USA) and Cell Tracker Deep Red Dye (cat. no.: C34565, Thermo Fisher Scientific, Grand Island, CA, USA), respectively. After 24 h of co‐culture, the cells were analyzed by fluorescence microscopy or flow cytometry. The phagocytosis ratio was calculated as the percentage of GFP‐labeled cells within the F4/80 population. For the cytotoxicity analysis, the bioluminescence was detected according to the instructions of the ONE‐Glo™ Luciferase Assay System (cat. no.: E6110, Promega, Wisconsin, USA). The tumor cell lysis ratio (%) was calculated using the following formula: (Sample signal‐tumor cell alone signal)/(Tumor cell alone signal‐background signal) ^*^100. The migration capability, adhesion ability, and tumoricidal activity of CAR‐HMs were also examined in transwell model and 3D tumor spheroids. Related information was shown in the Supporting Information.

### Animal Studies

Female C57BL/6J mice (6–8 weeks old) and female BALB/c nude mice (6–8 weeks old) were purchased from the Model Animal Research Center of Nanjing University, China, and raised under specific pathogen‐free conditions (SY2020003). The animal research was approved by the Animal Welfare and Ethics Committee of Nanjing University (IACUC‐2112012) and the maximal tumor size did not exceed 2000 mm^3^. PBS (100 µL) containing colorectal cancer cells (MC38^CEA^, MC38^CEA‐Luci^ or HT29^Luci^) was subcutaneously injected into corresponding C57BL/6J mice or BALB/c nude mice to generate ectopic CRC models.

To examine the biodistribution of CAR‐HMs in vivo, FcγRI‐CAR‐HMs (1 × 10^7^) or Ctr‐CAR‐HMs (1 × 10^7^) labeled with DiRIodide (cat. no.: HY‐D1048, Cy7 DiC18, MedChemExpress, Monmouth Junction, NJ, USA) were intravenously injected into MC38^CEA^ tumor bearing mice on day 8 post tumor cell inoculation. The IVIS Spectrum system (PerkinElmer) was applied to capture images of mice and their organs at indicated time points. Flow cytometry analysis and fluorescence microscope were used to assess the existence of mCherry^+^ CAR‐HMs in the tumor tissues. To evaluate the in vivo persistence of CAR‐HMs, FcγRI‐CAR‐HMs (1 × 10^7^) were injected into healthy mice via the tail vein. The proportions of CD45^+^F4/80^+^mCherry^+^ cells in the blood and various organs were subsequently analyzed at different time points using flow cytometry. To assess the efficacy of AP1903 in eliminating FcγRI‐CAR‐HMs in vivo, FcγRI‐CAR‐HMs (1 × 10^7^) were administered intravenously to mice on day 8 post tumor cell inoculation. AP1903 was subsequently injected intraperitoneally at a dose of 5 mg kg^−1^ on day 11 and 14 post tumor cell inoculation. Tumor growth rate, survival rate, and the presence of FcγRI‐CAR‐HMs in various organs were evaluated.

To evaluate the anti‐tumor effect of FcγRI‐CAR‐HMs, FcγRI‐CAR‐HMs (1 × 10^7^) or Ctr‐CAR‐HMs (1×10^7^) were intravenously injected on day 8 post tumor cell inoculation. In some cases, mice were co‐treated with anti‐PD‐1 antibody (10 mg kg^−1^ on days 9, 12, and 15 post‐tumor implantation) via intraperitoneal injection. Bioluminescence imaging was conducted on mice at specified time points to monitor tumor growth. Tumor volume was calculated using the formula: (length×width^2^)/2. Tumor tissues were excised, weighed, and used for H&E staining, immunopathological staining, flow cytometry, and ELISA assay on day 28 post tumor cell inoculation. Blood was harvested for flow cytometry and CEA examination. Body weight change during treatment, biochemical indicator assay (Jiancheng Bioengineering, Nanjing, China), and histological examination were performed to evaluate the in vivo safety of FcγRI‐CAR‐HMs. Detailed methods are provided in the Supporting Information.

### Statistical Analysis

Data were presented as Mean ± SD. Data were analyzed using GraphPad Prism 8.3.0 (GraphPad Software Inc. La Jolla, CA, USA), which included normality and homogeneity tests to validate parametric tests. Once normal distribution and homogeneity of variance were confirmed, the appropriate tests were selected: unpaired student's t‐test (two tailed) for two‐group comparisons, one‐way ANOVA with Dunnett's multiple comparisons test or Tukey's multiple comparisons test for multiple group comparisons, two‐way ANOVA with Sidak's multiple comparisons test for interactions. For data that did not meet the normality criterion, non‐parametric tests were applied, including the Mann‐Whitney U test (two‐tailed), or Kruskal‐Wallis test followed by Dunnett's multiple comparisons test. In cases of normal distribution with unequal variances, the t test with Welch's correction (two tailed), or Brown‐Forsythe and Welch ANOVA with Tamhane's T2 multiple comparisons test were used. The log‐rank (Mantel‐Cox) test was used to analyze the statistical significance of difference for survival analysis. Statistical significance was considered when P < 0.05, and “ns” indicates no significance.

## Conflict of Interest

The authors declare no conflict of interest.

## Author Contributions

H.Z., C.J.N., and Z.J.F. conceptualized and designed this study. G.C.C. performed most experiments. H.F.L., D.Y., F.Y., M.Y.N., and S.X.D. performed partial experiments. G.C.C. finished the acquisition and analysis of data. G.C.C. prepared figures, performed the statistical analysis, and wrote original draft. H.Z., C.J.N., and Z.J.F. reviewed and supervised the manuscript. All authors read and approved the final manuscript.

## Supporting information



Supporting Information

## Data Availability

The data that support the findings of this study are available in the supplementary material of this article.
