# Supplementary material for: Lifespan‐Regulated CAR‐Macrophages from Myeloid Progenitors for Enhanced Colorectal Cancer Therapy
Source: Adv Sci (Weinh). 2025 Aug 22;12(43):e17677. doi: 10.1002/advs.202417677 (PMC12631822; doi:10.1002/advs.202417677)
Supplement: Supplementary file 1 — Supporting Information [file ADVS-12-e17677-s001.docx]

Supporting Information

**Lifespan-regulated CAR-macrophages from Myeloid Progenitors for Enhanced** **Colorectal Cancer Therapy**

***Chuancheng Gao^1^, Fangling Hong^1^, Yao Dong^1^, Yong Fu^1^, Yunong Ma^1^, Xuedi Sun^1^, Junfeng Zhang^1^*, Jiangning Chen^1, 2^*, Zhen Huang^1,3^****

**^1^State Key Laboratory of Pharmaceutical Biotechnology, School of Life Sciences, Nanjing University, Nanjing, Jiangsu 210023, China**

**^2^State Key Laboratory of Analytical Chemistry for Life Sciences, Nanjing University, Nanjing, Jiangsu 210023, China**

^3^NJU Xishan Institute of Applied Biotechnology, Xishan District, Wuxi, Jiangsu, 214101, China

***Correspondence: Junfeng Zhang (jfzhang@nju.edu.cn), Jiangning Chen (jnchen@nju.edu.cn), or Zhen Huang (zhenhuang@nju.edu.cn)**

**Supplementary methods**

*qRT-PCR analysis:* Total RNA was isolated from cells under various treatment conditions using TRIzol reagent and reverse transcribed into cDNA with the cDNA Reverse Transcription Kit (cat. no.: R223-01, Vazyme, Nanjing, China). Quantitative real-time PCR (qRT-PCR) was performed using the StepOne™ Real-Time PCR System (Thermo Fisher Scientific, Grand Island, CA, USA) to measure the relative mRNA levels of the target genes. β-actin served as an internal control. The primer sequences used in the qRT-PCR experiments are listed in the Supplementary Table 4.

*Western blotting assay:* Cell samples were collected and lysed with RIPA lysis buffer containing protease inhibitors (cat. no.: P0013K, Beyotime, Shanghai, China), followed by protein extraction. Protein concentration was determined using the Pierce BCA Protein Assay Kit (cat. no.: 23227, Thermo Fisher Scientific, Grand Island, CA, USA). Sodium dodecyl sulfate-polyacrylamide gel electrophoresis was conducted, and then the samples were transferred onto polyvinylidene fluoride films. Proteins were separated by sodium dodecyl sulfate-polyacrylamide gel electrophoresis (SDS-PAGE) and transferred onto PVDF membranes. Membranes were exposed to primary antibodies, washed, and then incubated with corresponding secondary antibodies. GAPDH was used as an internal control. Detailed information on the antibodies used is provided in the Supplementary Table 5.

*Flow cytometry analysis:* The cell suspensions from blood, spleen, liver, bone marrow, lung, and tumor tissues were filtered through Nylon cell strainers (70 μm, Falcon, CA, USA). Red blood cells were lysed to isolate primary cells. These primary cells, along with cell lines, HPCs, and their derived macrophages, were rinsed with PBS and blocked with Fc-antibody diluted in PBS containing BSA (1%) on ice for 10 minutes. For cell surface molecule staining, cells (1×10^6^) were incubated with fluorescence-labeled antibodies for 30 minutes on ice, followed by washing with BSA (1%). 7-AAD staining was employed to differentiate live and dead cells. Intracellular staining was performed as follows: Tumor leukocytes were initially incubated with Fc-blocking antibodies to minimize nonspecific binding, followed by surface marker labeling using specific antibodies. Cells were subsequently fixed and permeabilized using BD Cytofix/Cytoperm solution (BD Biosciences, San Jose, CA, USA) to enable intracellular detection of IFN-γ, Ki-67, and Granzyme B with corresponding antibodies. For IFN-γ analysis, cells were pretreated with Cell Activation Cocktail containing Brefeldin A (BioLegend, San Diego, CA, USA) for 4 hours to promote cytokine retention prior to surface staining. Cell viability was assessed using the Zombie Violet™ Fixable Viability Kit (BioLegend) to exclude dead cells from analysis. Flow cytometry experiments were performed on an Attune NxT device (Thermo Fisher Scientific, CA, USA) and analyzed using FlowJo V10 software (BD Biosciences, Ashland, OR, USA). Detailed information on the antibodies used can be found in the Supplementary Table 5.

*Cell migration and adhesion assay:* For the trans-endothelial migration assay of FcγRI-CAR-HMs or Ctr-CAR-HMs, HUVECs (2×10^5^) were first seeded in the upper chamber of 24-well Transwell (pore size, 8 µm; BD Biosciences) precoated with gelatin (0.1%). After 12 hours culture, HUVECs were stimulated with IL-1α (5 ng/mL, cat. no.: 200-01A, PeproTech, Rocky Hill, NJ, USA) for 4 hours, after which the cells were washed three times with PBS to remove IL-1α. Both the lower and upper chambers were then filled with RPMI 1640 medium containing FBS (10%), mouse M-CSF (20 ng/mL), mouse GM-CSF (20 ng/mL), and CEA (100 ng/mL). Macrophages (5×10^4^) were serum-starved for 3 hours and then seeded in the upper chamber. Under some circumstances, Batimastat (BB-94, 10 nм, cat. no.: HY-13564, MedChemExpress, Monmouth Junction, NJ, USA), a broad-spectrum matrix metalloproteinase inhibitor, was added into the upper chambers. After 12 hours, non-migrated cells were removed, and cells that had migrated through the porous membrane were fixed, stained with crystal violet, and counted under a microscope.

For the adhesion assay involving FcγRI-CAR-HMs or Ctr-CAR-HMs, HUVECs (1×10^6^ cells/well) were seeded into 6-well plates and cultured until approximately 90% confluent. The cells were then stimulated with IL-1α (5 ng/mL) for 4 hours. Following stimulation, the cells were washed three times with PBS to remove residual IL-1α. FcγRI-CAR-HMs or Ctr-CAR-HMs (2*10^5^ cells per well pre-stimulated with CEA (100 ng/mL) for 48 hours) were then added to the wells and incubated for 1 hour. After incubation, the culture supernatant was removed, unattached cells were washed off with PBS, and the adhered macrophages were dissociated using cell dissociation solution (cat. no.: 13151014, Thermo Fisher Scientific, Grand Island, CA, USA). The number of collected cells was counted, and the number of adhered macrophages was determined by subtracting the number of HUVECs.

*Macrophages’ infiltration of 3D tumor spheroids:* 3D tumor spheroids were generated by culturing tumor cells in a Corning 96-well ultra-low attachment plate (cat. no.: CON7007, Corning, New York, USA). Specifically, MC38^CEA^ cells (5×10^3^) were suspended in a 96-well ultra-low attachment plate containing culture medium. The cultures were maintained at 37°C in CO_2_ (5%) until cell clusters formed, typically within 5 to 7 days. Each cell cluster was then transferred to a single well in a 24-well plate for further analysis.

To assess the adhesive infiltration ability of FcγRI-CAR-HMs or Ctr-CAR-HMs on the tumor spheroids, macrophages (2×10^5^) were added to the wells containing tumor spheroids. The co-cultures were maintained for 48 hours, and the 3D tumor spheroids were imaged using a Zeiss LSM880 confocal microscope (CARL ZEISS, Oberkochen, German).

To analyze the lytic ability of FcγRI-CAR-HMs on the tumor spheroids, macrophages (2×10^5^) were co-cultured with the tumor spheroids for 0.5 to 5 days, and the 3D tumor spheroids were imaged at indicated time points and the protein was extracted from tumor spheroids 5 days post to co-culture for western blotting assay.

*Transcriptome sequencing and bioinformatic analysis:* Total RNA was extracted from FcγRI-CAR-HMs, and Ctr-CAR-HMs using TRIzol reagent, following the manufacturer's protocol. To prevent genomic DNA contamination, RNA samples were treated with RNase-free DNase. Transcriptome sequencing was conducted by BGI Genomics Co., Ltd (Shenzhen, China). Differentially expressed genes (DEGs) were identified using the criteria: fold change ≥1.5 and P value < 0.05.

To explore the primary biological functions of DEGs from FcγRI-CAR-HMs or Ctr-CAR-HMs, Kyoto Encyclopedia of Genes and Genomes (KEGG) pathway analysis was performed using KOBAS 3.0 software (<http://kobas.cbi.pku.edu.cn/kobas3/>), with significance defined as P value < 0.05. Gene set enrichment analysis (GSEA) was carried out using software provided by the Massachusetts Institute of Technology. The normalized enrichment score (NES) quantified the enrichment magnitude, and the false discovery rate (FDR) quantified statistical significance.

*ELISA assays:* MC38^CEA^ (100 mg) tumors were homogenized in cold PBS (500 μL) using a rotor stator (5 mm beads, 60 s × 2 replications at 60 Hz, Tissuelyser-24, Jingxin Industrial Development Co., Ltd., Shanghai, China) and then centrifuged at 12,000 rpm for 10 minutes at 4 °C to obtain the supernatants. TAMs isolated from MC38^CEA^ tumors were lysed and the protein concentrations of lysate were determined using a BCA protein assay (Sangon Biotech). Blood samples from MC38^CEA^ tumors bearing mice were centrifuged at 3,000 rpm for 20 minutes at 4 °C to obtain serum. The supernatant of MC38^CEA^ cells and FcγRI-CAR-HMs with different treatments were collected. The levels of cytokines were determined using ELISA kits (Thermo Fisher) following the manufacturer's instructions and further normalized to total volume (supernatants and serum) or mg total protein (lysates).

*Pathological Analyses:* Mouse organs (heart, liver, spleen, lung, and kidney) and tumor tissues were fixed in formalin, embedded in paraffin, and stained with hematoxylin and eosin (H&E). For macrophage infiltration assays, frozen tumor sections were stained with DAPI and visualized under a ZEISS LSM 980 confocal microscope (CARL ZEISS, Oberkochen, German). Immunohistochemical staining was performed on deparaffinized sections after antigen retrieval and blocking with PBS containing BSA (5%). Primary antibodies against CD8, Perforin, Granzyme B, and Cleaved caspase-3 were applied, followed by biotinylated secondary antibodies and peroxidase-labeled streptavidin. Negative controls included sections treated only with secondary antibodies. Detailed information on the antibodies used is provided in the Supplementary Table 5.

**Supplementary Figures**


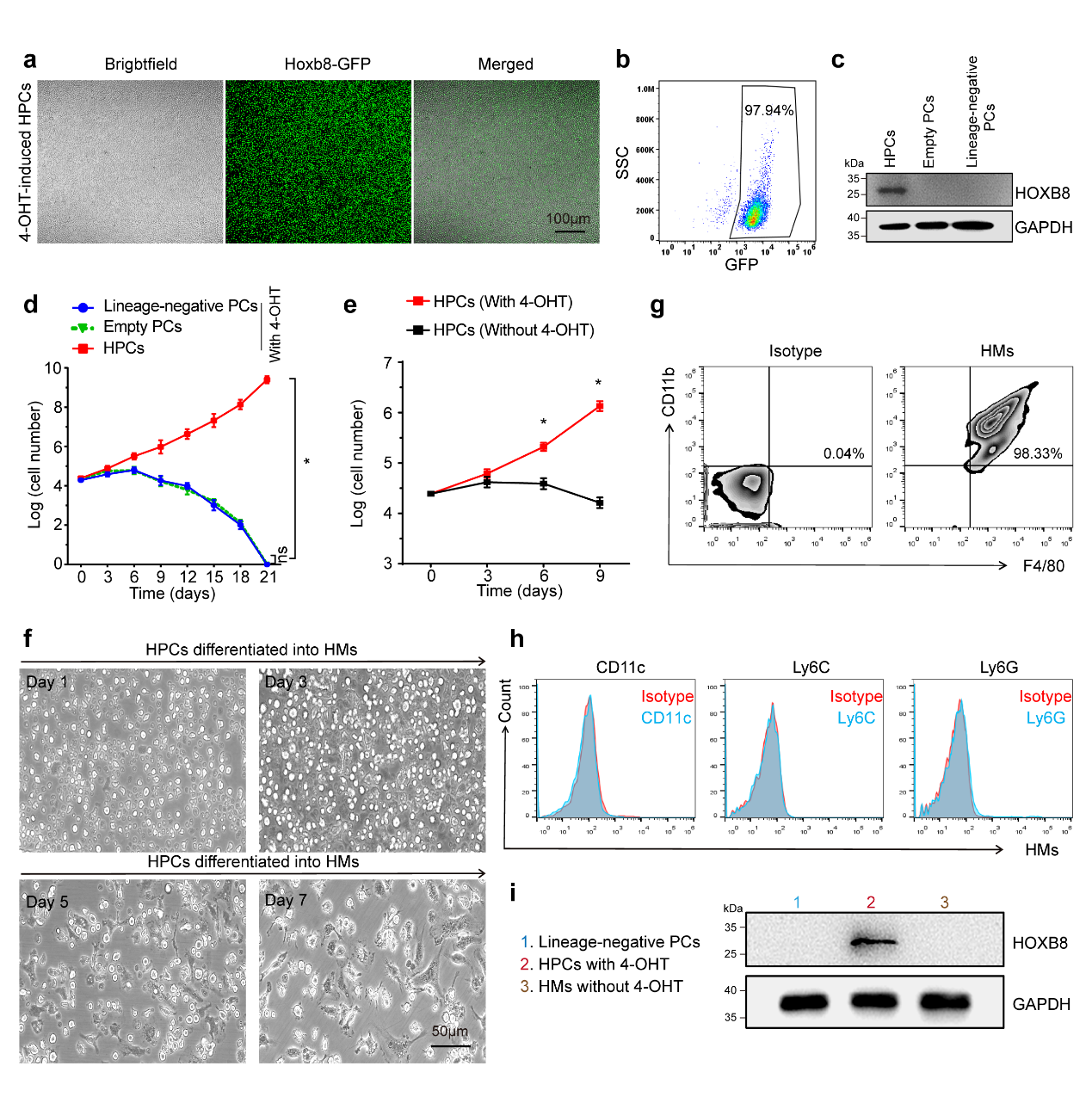


**Supplementary Figure 1. Construction of proliferative HPCs and induced their differentiation into macrophages.** (a-c) Lineage-negative progenitors were transduced with the retrovirus encoding GFP and estrogen receptor-fused Hoxb8. Hoxb8 expression by progenitors was examined by fluorescence microscopy, flow cytometry and western blotting. Scale bar, 100 μm. (d-e) Proliferative properties of HPCs in the presence of 4-hydroxytamoxifen (4-OHT) or withdrawal of 4-OHT. (f) The morphological changes of HPCs in the absence of 4-OHT and the addition of M-CSF (20 ng/mL) and GM-CSF (20 ng/mL). Scale bar, 50 μm. (g-h) Flow cytometry analysis of neutrophil marker (Ly6G), monocyte marker (Ly6C), DC marker (CD11c) and macrophage markers (F4/80 and CD11b) in macrophages differentiated from HPCs. (i) Protein expression of Hoxb8 in HPCs and HMs were examined by western blotting. n = 3 biologically independent samples for panels (b, d-g, h). Data are shown as means ± SD. Statistical analyses were performed using two-way ANOVA test with Sidak's multiple comparisons test for panels (d, e). Significance: *P < 0.05, ns, not significant. ANOVA, analysis of variance.


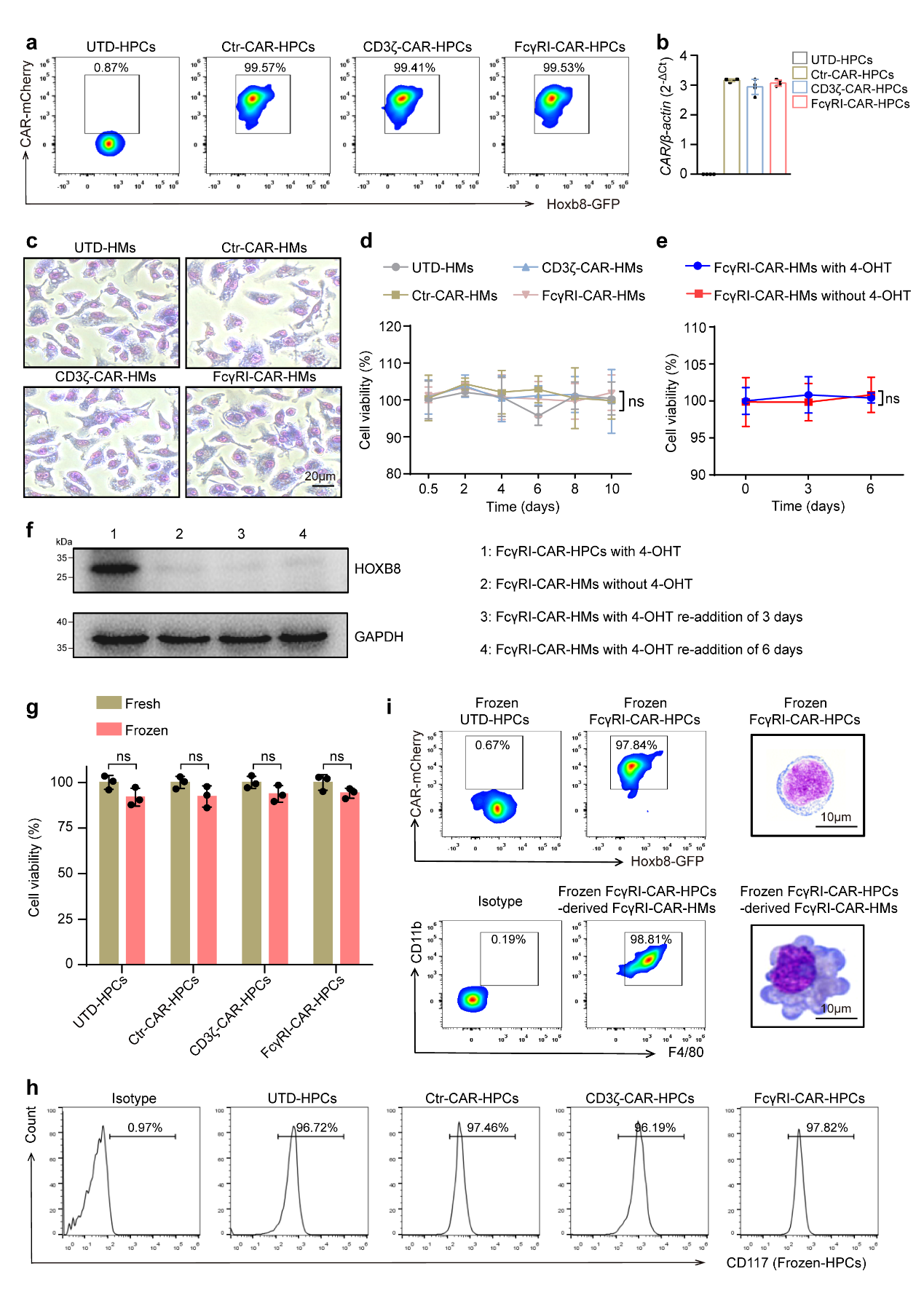


**Supplementary Figure 2. The construction and cryopreservation of CAR-HPCs.** (a) Flow cytometry analysis of HPCs co-expressing both Hoxb8-GFP and CAR-mCherry. (b) qRT-PCR analysis of CAR mRNA levels in different types of CAR-HPCs. n = 4 biologically independent samples. (c-d) The cell morphology and viability of differentiated CAR-HMs were characterized by Diff-Quick staining and CCK-8 (n = 4 biologically independent samples). Scale bar, 20 μm. (e) The cell viability of FcγRI-CAR-HMs with or without 4-OHT (1 µм) was examined by CCK-8 assay. n = 4 biologically independent samples. (f) Protein expression of Hoxb8 in FcγRI-CAR-HPCs and FcγRI-CAR-HMs (with 4-OHT re-addition) were examined by western blotting. (g-h) The viability and stemness of CAR-HPCs, after 6 months of freezing in liquid nitrogen and subsequent thawing, were assessed using the CCK-8 assay and flow cytometry. n = 3 biologically independent samples. (i) The CAR expression, macrophage differentiation efficiency (CD11b and F4/80 double positive) and cell morphology of cryopreserved FcγRI-CAR-HPCs were examined by flow cytometry and Wright-Giemsa staining. Scale bar, 10 μm. Results in panels (a), (h) and (i) are representative plots from three biological replicates, processed using FlowJo. Data are shown as means ± SD. Statistical analyses were performed using two-way ANOVA test with Sidak's multiple comparisons test for panels (d), two-way ANOVA test with Bonferroni's multiple comparisons test (e), two-tailed unpaired t test for panel (g). ns, not significant.


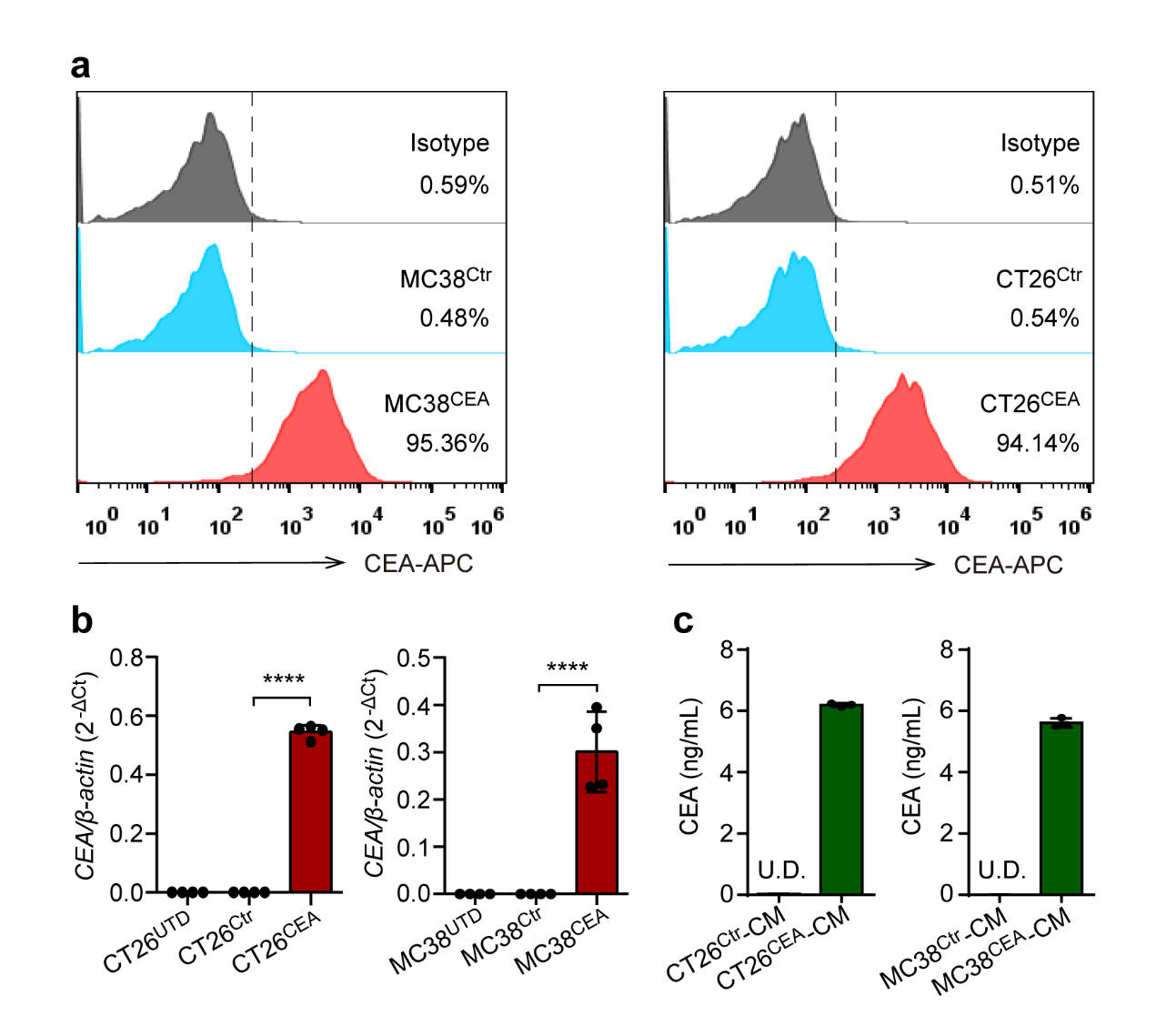


**Supplementary Figure 3. The examination of CEA expression in CRC stable cell lines.** (a) Flow cytometry analysis of CT26 and MC38 cells expressing CEA. Results in panel (a) are representative plots from three biological replicates, processed using FlowJo software. (b) qRT-PCR assay of CEA mRNA levels in MC38^CEA^ and CT26^CEA^ cells. n = 4 biologically independent samples. (c) ELISA assay of CEA protein levels in MC38^CEA^ and CT26^CEA^ cells. n = 3 biologically independent samples. Data are shown as means ± SD. Statistical analysis was performed using one-way ANOVA test with Tukey’s multiple comparisons test for panel (b). Significance: ****P < 0.0001, U.D., undetected.


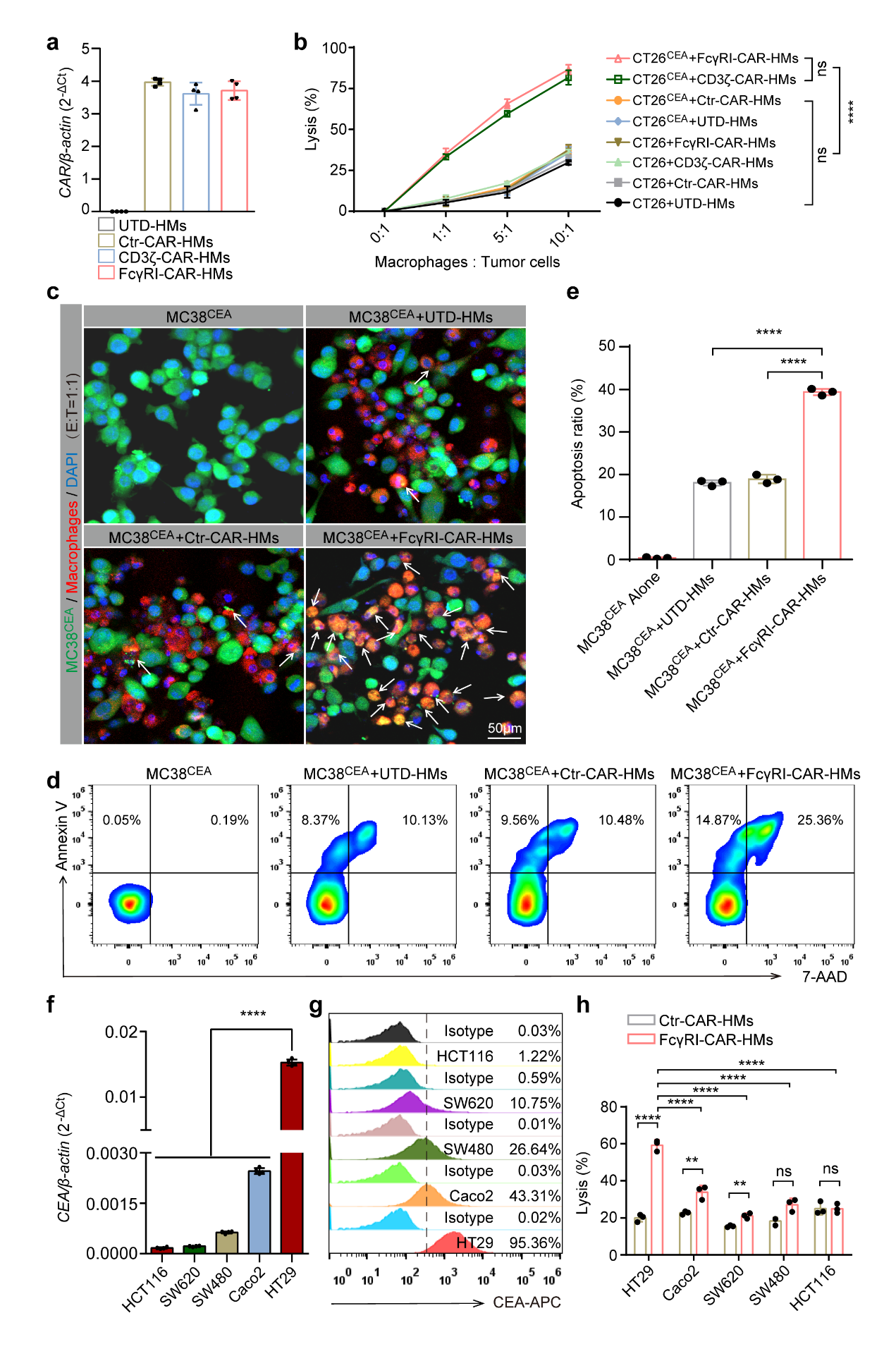


**Supplementary Figure 4. The assessment of anti-tumor capability of FcγRI-CAR-HMs towards colorectal cells.** (a) qRT-PCR analysis of CAR expression in CAR-HMs. n = 4 biologically independent samples. (b) Different types of CAR-HMs were co-cultured with CT26^CEA-Luci^ cells at different ratios for 24 hours. CAR-HMs mediated tumor cells lysis was calculated by examining the bioluminescence intensity. n = 3 biologically independent samples. (c) Confocal images showing phagocytosis of MC38^CEA^ tumor cells (green) by CAR-HMs (red) at the Macrophages: Tumor cells ratio = 1:1 (arrows indicate phagocytosis events). Scale bar, 50 μm. (d-e) Flow cytometry analysis of FcγRI-CAR-HMs mediated apoptosis of MC38^CEA^ tumor cells after co-culture for 24 hours. n = 3 biologically independent samples. (f-g) qRT-PCR and flow cytometry analysis of CEA expression in human colorectal cancer cell lines. n = 4 biologically independent samples. (h) FcγRI-CAR-HMs were co-cultured with different colorectal cancer cell lines at the ratio of 5:1 for 24 hours. CAR-HMs mediated tumor cell lysis was calculated by examining the bioluminescence intensity (n = 3 biologically independent samples). Results in panels (d) and (g) are representative plots from three biological replicates, processed using FlowJo software. Data are shown as means ± SD. Statistical analysis was performed using one-way ANOVA test with Tukey’s multiple comparisons test for panels (b, e, h), one-way ANOVA test with Dunnett's multiple comparisons test for panel (f), two-tailed unpaired t test for panel (h) except SW480 group and Mann Whitney test for panel (h, SW480 group). Significance: **P < 0.01, ****P < 0.0001, ns, not significant.


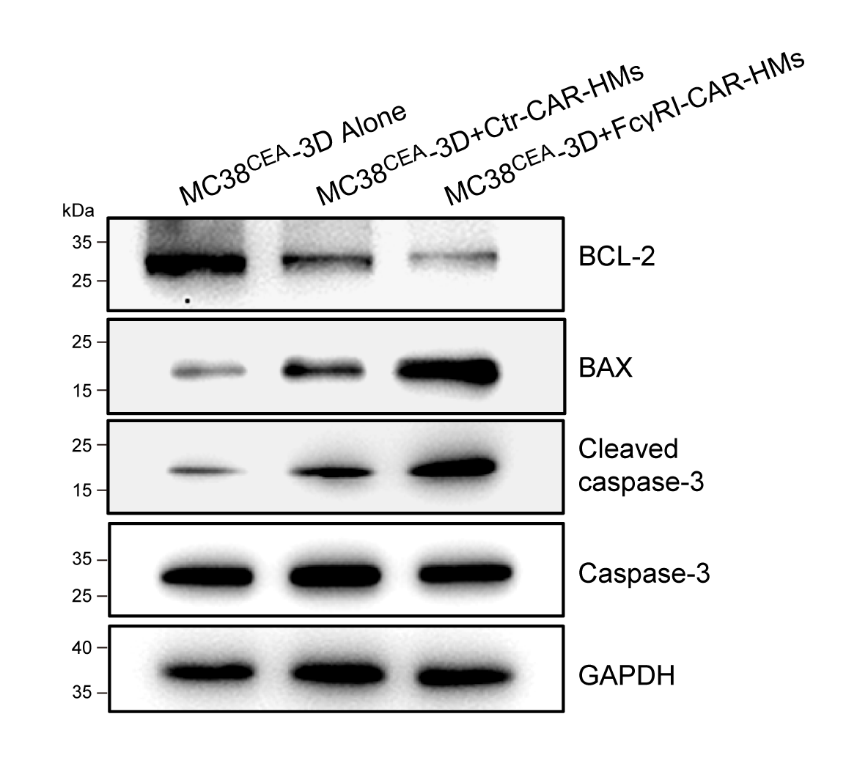


**Supplementary Figure 5. Western blotting of related apoptotic proteins in MC38^CEA^ 3D tumor spheroids.** FcγRI-CAR-HMs were co-cultured with MC38^CEA^  3D tumor spheroids for 5 days, and the expression of BAX, BCL-2, and Caspase-3 were detected by western blotting.


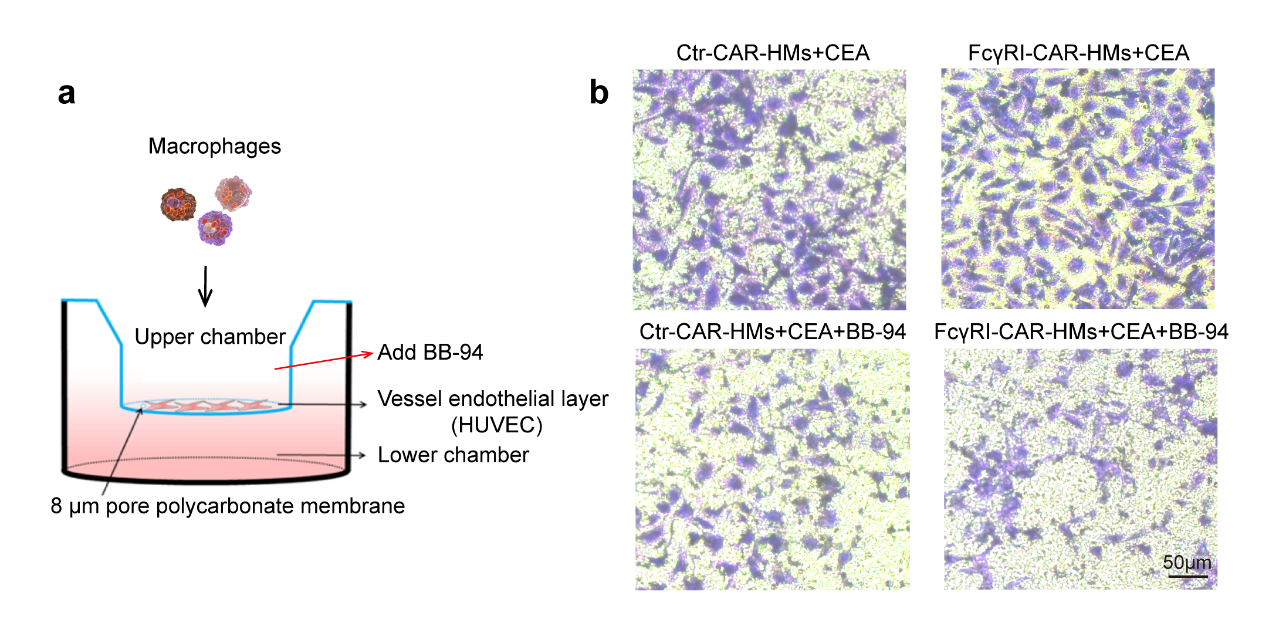


**Supplementary Figure 6. CEA-mediated FcγRI-CAR-HMs activation enhanced their migration capability.** (a) Schematic diagram of the trans-endothelial migration model. HUVECs (2×10^5^) were first seeded in the upper chamber of 24-well Transwell (pore size, 8 µm; BD Biosciences) precoated with gelatin (0.1%). After 12 hours culture, HUVECs were stimulated with IL-1α (5 ng/mL) for 4 hours. Then, FcγRI-CAR-HMs (5×10^4^) were seeded in the upper chamber. Under some circumstances, Batimastat (BB-94, 10 nм) was added to the upper chambers. (b) After 12 hours, FcγRI-CAR-HMs migrating through the porous membrane were fixed, stained with crystal violet. n = 3 biologically independent samples. Representative images are shown. Scale bar, 50 μm.


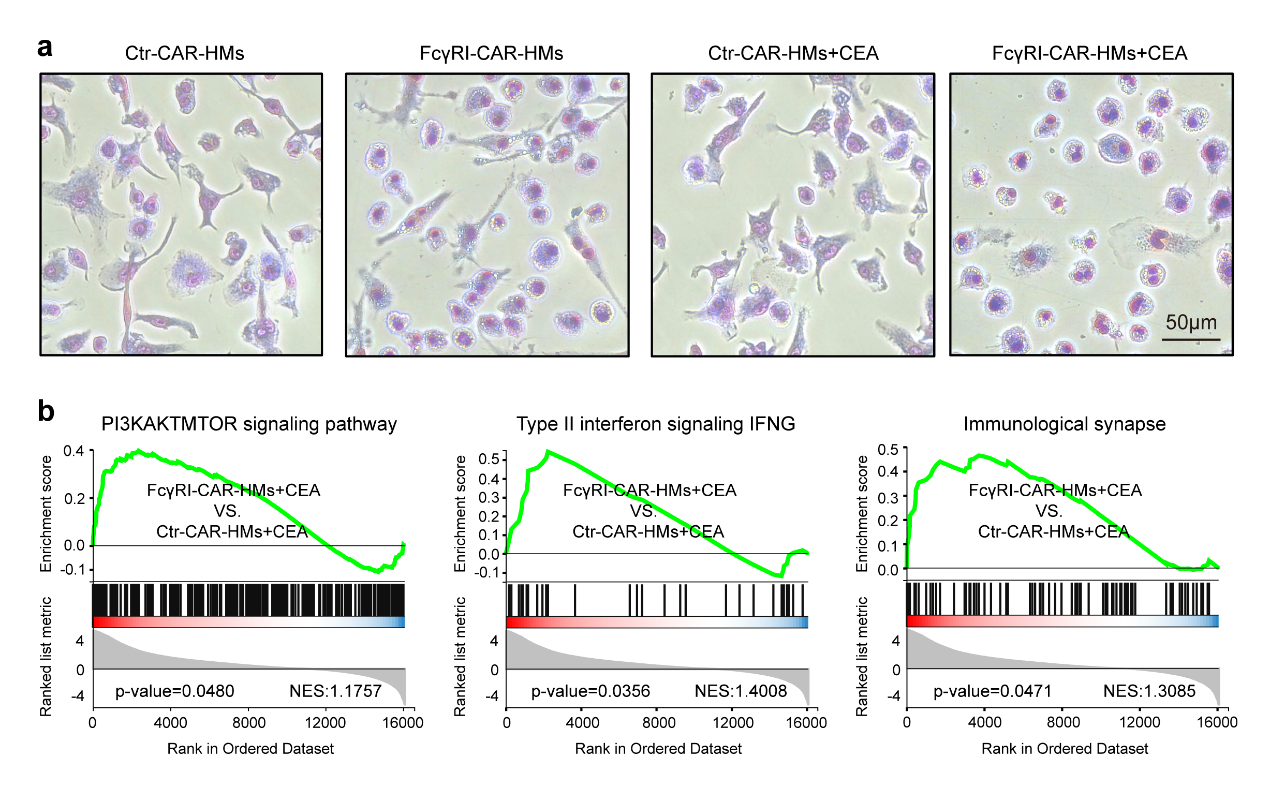


**Supplementary Figure 7. Morphological and transcriptional characterization of FcγRI-CAR-HMs following CEA treatment.** (a) The cell morphology of FcγRI-CAR-HMs and Ctr-CAR-HMs treated with CEA (100 ng/mL) for 48 hours. Scale bar, 50 μm. (b) GSEA enrichment analysis demonstrating the enrichment of upregulated genes in PI3KAKT MTOR signaling pathway, Type II interferon signaling IFNG and Immunological synapse. n = 3 biologically independent samples. Representative images for panel (a) are shown.


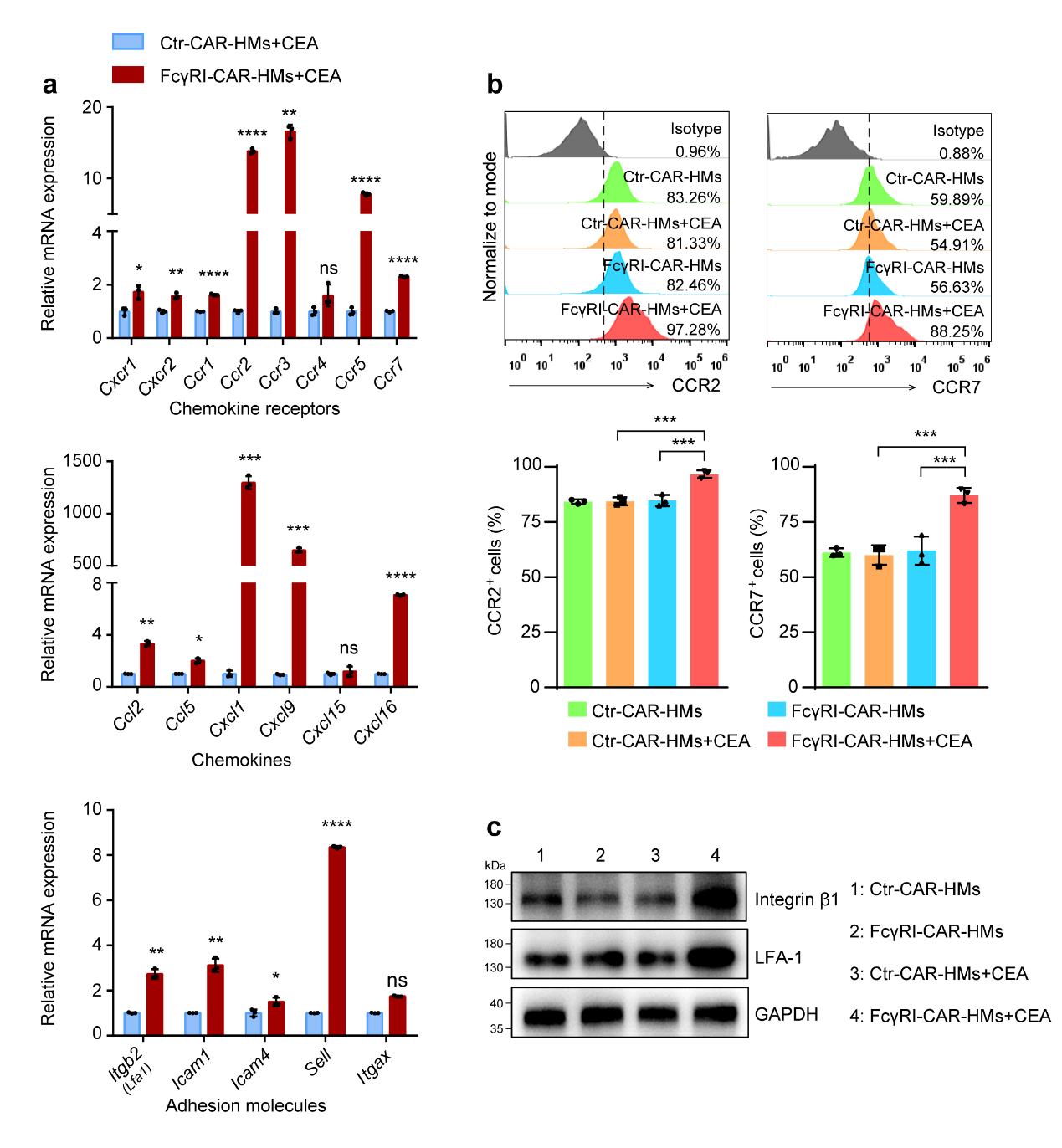


**Supplementary Figure 8. Upregulation of adhesion molecules, chemokines and chemokine receptors of FcγRI-CAR-HMs following CEA treatment.** (a) The mRNA levels of adhesion molecules, chemokines and chemokine receptors in FcγRI-CAR-HMs and Ctr-CAR-HMs treated with CEA (100 ng/mL) for 48 hours. (b-c) The expression of chemokine receptors (CCR2 and CCR7) and adhesion molecules (Integrin β1 and LFA-1) in above mentioned treated Ctr-CAR-HMs and FcγRI-CAR-HMs was detected by flow cytometry or western blotting, respectively. n = 3 biologically independent samples for panels (a-b). Data are shown as means ± SD. Statistical analysis was performed using two-tailed unpaired t test for panel (a: *Cxcr1*, *Cxcr2*, *Ccr1*, *Ccr2*, *Ccr5*, *Ccr7*, *Cxcl15*, *Cxcl16*, *Icam4*, *Sell*), two-tailed unpaired t test with Welch’s correction for panel (a: *Ccr3*, *Ccl2*, *Ccl5*, *Cxcl1*, *Cxcl9*, *Itgb2*, *Icam1*), Mann Whitney test for panel (a: *Ccr4*, *Itgax*) and one-way ANOVA test with Tukey’s multiple comparisons test for panel (b). Significance: *P < 0.05, **P < 0.01, ***P < 0.001; ****P < 0.0001, ns, not significant.


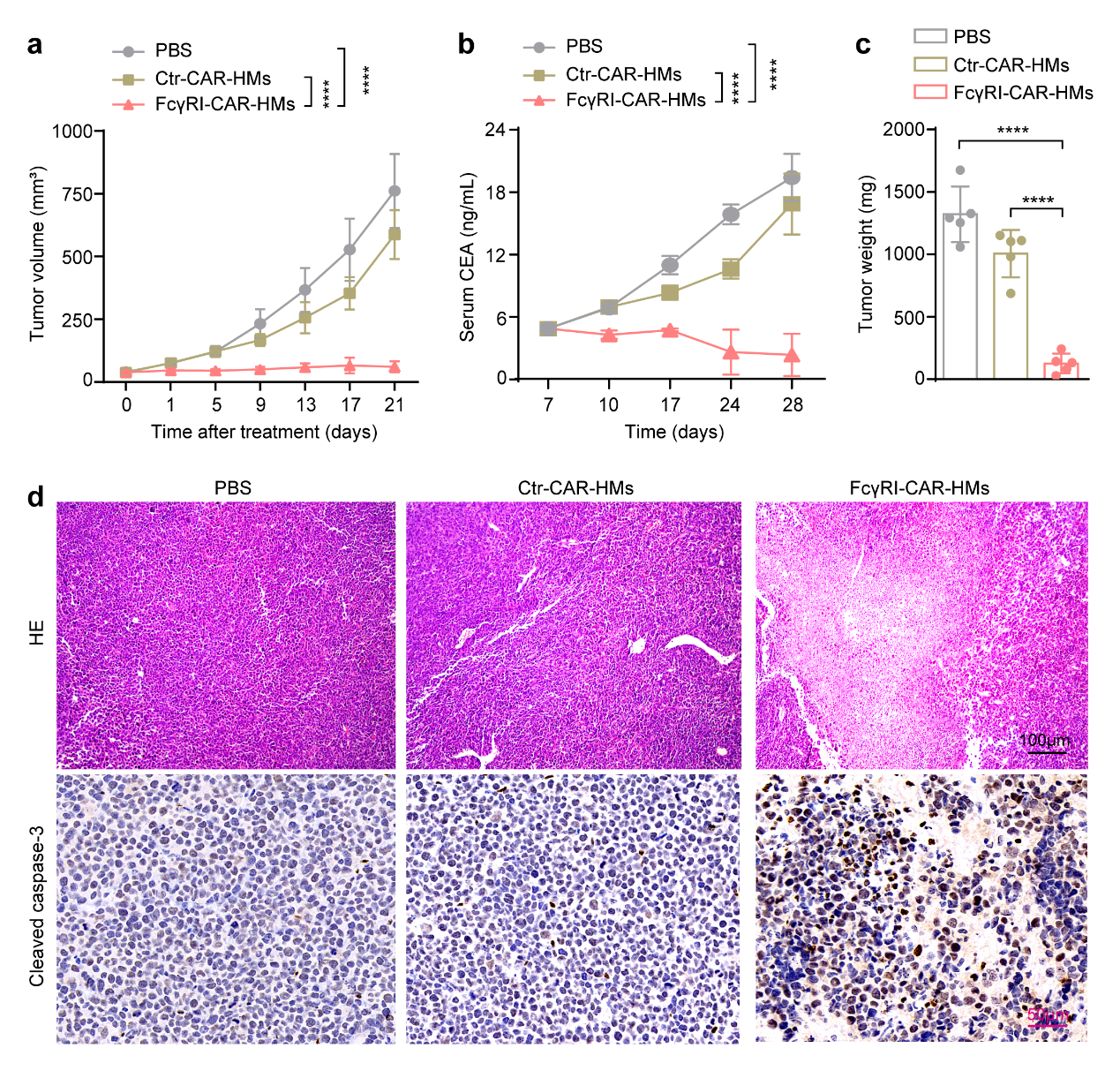


**Supplementary Figure 9. FcγRI-CAR-HMs suppressed the growth of MC38^CEA^ tumors.** (a) Tumor volume curves of MC38^CEA-Luci^ tumor bearing mice intravenously injected with different types of CAR-HMs (1×10^7^ cells per mouse) on day 8 post tumor cell inoculation. (b) The CEA levels in the serum from tumor bearing with above mentioned treatments at various time points were examined by ELISA assay. (c) Tumor weights in mice treated with different CAR-HMs on day 28 post tumor cell inoculation. (d) Representative H&E and immunohistochemical staining of Cleaved caspase-3 in MC38^CEA^ tumor tissues from mice with above mentioned treatments on day 28 post tumor cell inoculation. Scale bars are included in panel (d) for reference. Data are shown as means ± SD. Statistical analysis was performed using two-way ANOVA test with Sidak's multiple comparisons test for panels (a, b) and one-way ANOVA test with Tukey’s multiple comparisons test for panels (c). Significance: ****P < 0.0001.


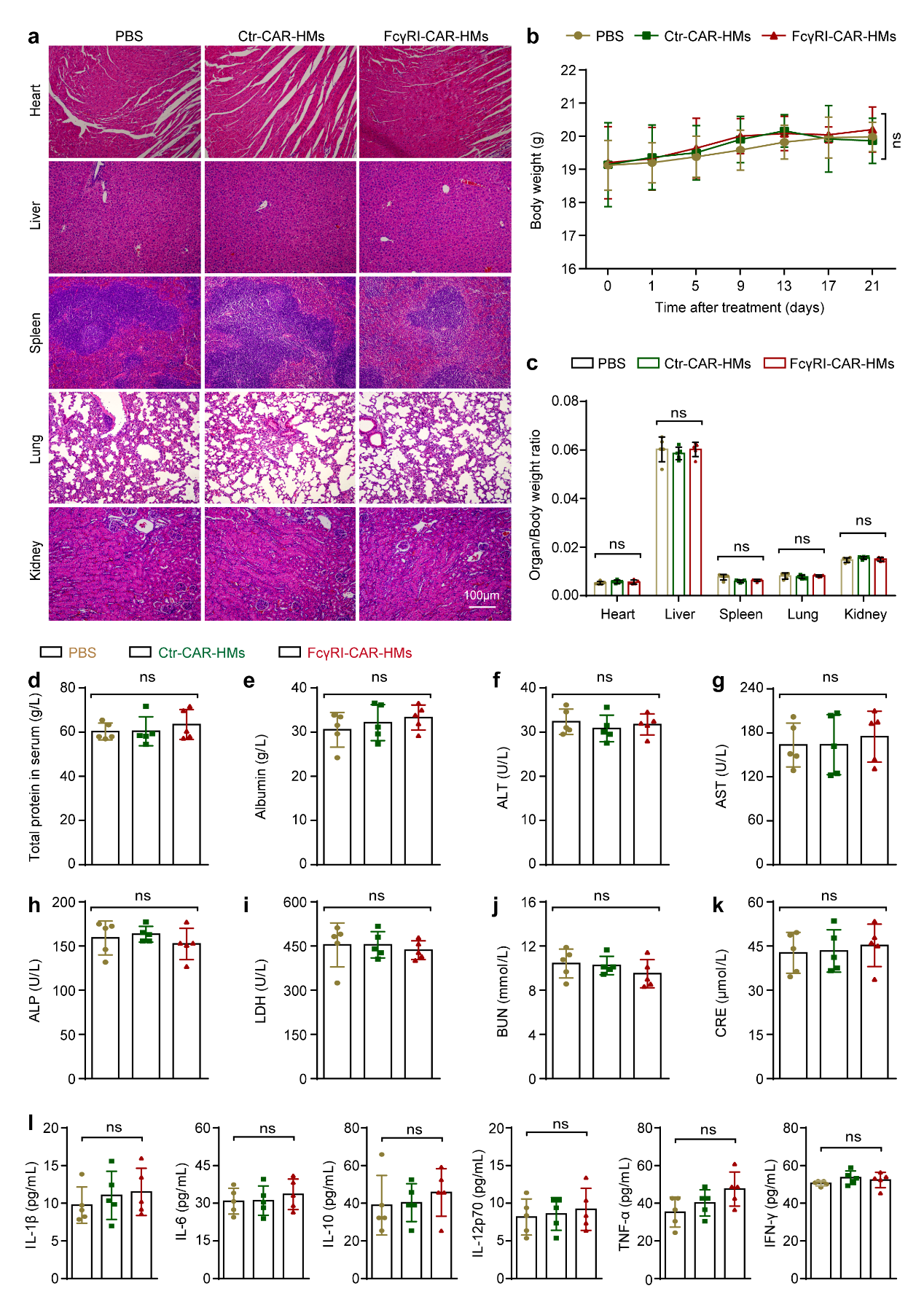


**Supplementary Figure 10. Safety assay in MC38^CEA^ tumor bearing mice after treatment with different types of CAR-HMs.** (a) Representative H&E staining of tissues sections harvested from heart, liver, spleen, lung and kidney of mice, after the injection of FcγRI-CAR-HMs or Ctr-CAR-HMs. Scale bar, 100 μm. (b-c) Changes in body weight and organ index (organ weight/body weight) of mice receiving above mentioned treatments. (d-k) The measurement of biochemical indices (total protein, albumin, alanine aminotransferase (ALT), aspartate aminotransferase (AST), alkaline phosphatase (ALP), lactate dehydrogenase (LDH), blood urea nitrogen (BUN), and creatinine (CRE)) in blood of mice with above-mentioned treatments. (l) The levels of cytokines were detected by ELISA in the serum from mice with CAR-HMs treatments. n = 5 mice per group. Data are shown as means ± SD. Statistical analysis was performed using two-way ANOVA test with Sidak's multiple comparisons test for panel (b), one-way ANOVA test with Tukey’s multiple comparisons test for panels (c-l) except panel (c, spleen index) and Kruskal-Wallis test with Dunnett's multiple comparisons test for panel (c, spleen index). ns, not significant.


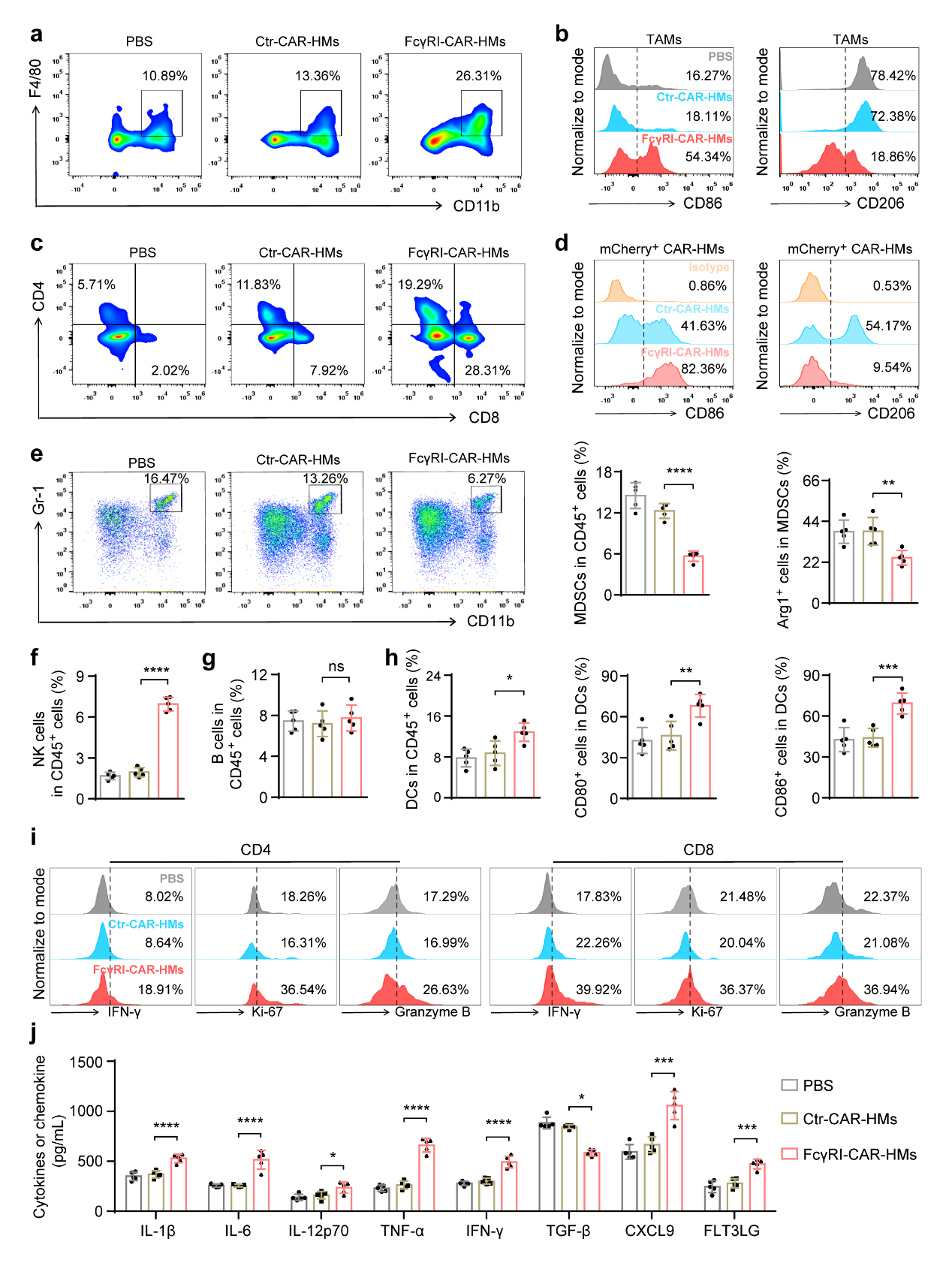


**Supplementary Figure 11. FcγRI-CAR-HMs treatment reshaped the immune cell landscape of MC38^CEA^ tumor bearing mice.** (a-i) The leukocyte suspensions from tumor tissues of mice on day 28 post tumor cell inoculation were analyzed by flow cytometry. The influence of FcγRI-CAR-HMs on (a-b) TAMs ratio and phenotype (CD86 and CD206), (c) CD4^+^ T cells and CD8^+^ T cells ratios, (d) CAR-HMs phenotypes, (e) ratios of MDSCs and Arg1^+^ MDSCs, (f) NK cells, (g) B cells, (h) DCs ratio and activated DCs (CD80^+^ and CD86^+^), (i) T cells activation and proliferation (percentage of IFN-γ^+^, ki-67^+^ and Granzyme B^+^ cells in CD4^+^ T cells and CD8^+^ T cells) was examined. (j) Cytokine and chemokine levels in MC38^CEA^ tumor tissues of mice after treated with different types of CAR-HMs were determined by ELISA on day 28 post tumor cell inoculation. n = 5 mice per group. Data are shown as means ± SD. Statistical analysis was performed using one-way ANOVA test with Tukey’s multiple comparisons test for panels (e-j) except panel (j, TGF-β) and Kruskal-Wallis test with Dunnett's multiple comparisons test for panel (j, TGF-β). Significance: *P < 0.05, **P < 0.01, ***P < 0.001, ****P < 0.0001, ns, not significant.


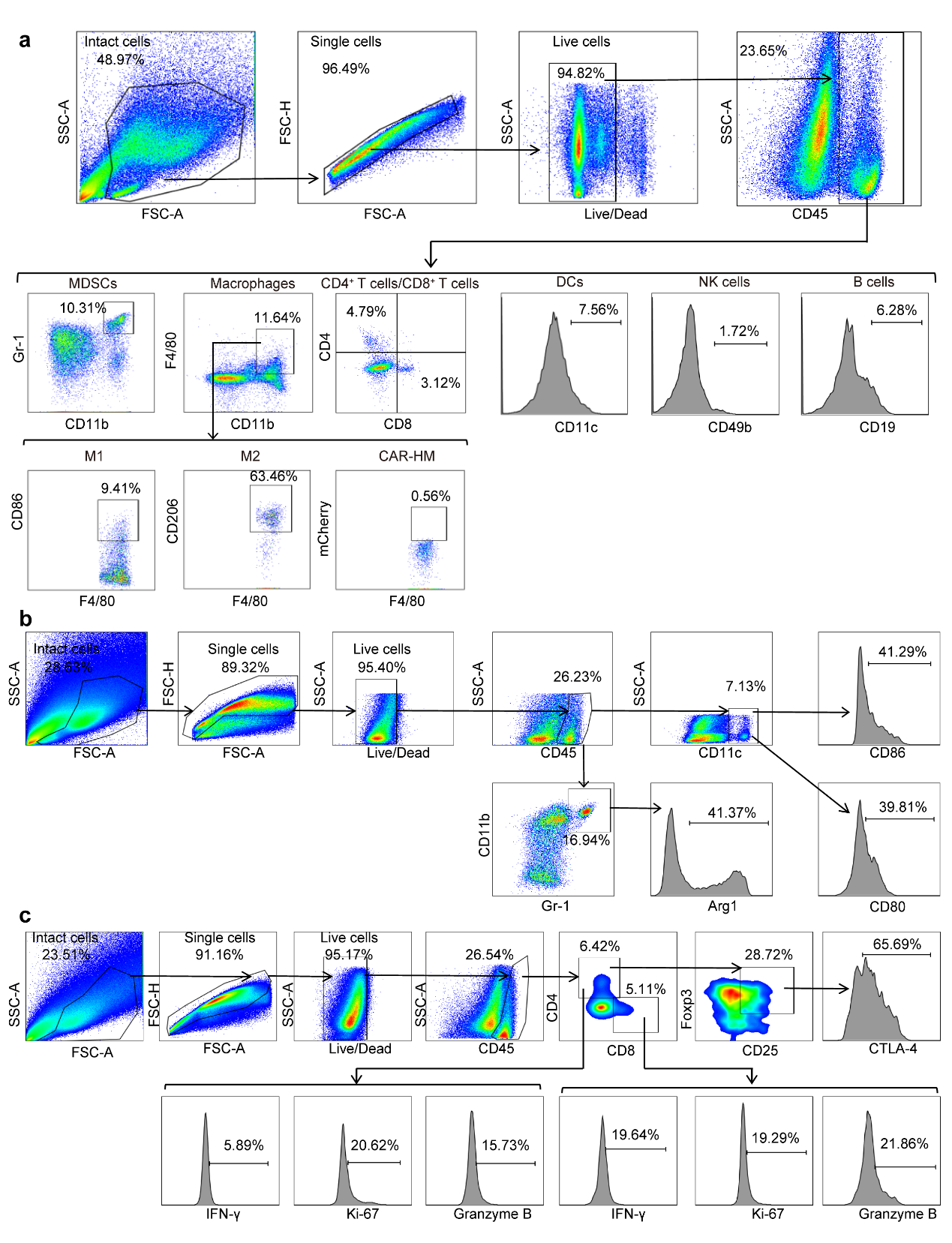


**Supplementary Figure 12.** **Gating strategies of cell populations in tumor used for flow cytometry analysis.** (a) CD4^+^ and CD8^+^ T lymphocytes, macrophages (CD11b^+^F4/80^+^), MDSCs (CD11b^+^Gr-1^+^), DCs (CD11c^+^), B cells (CD19^+^) and NK cells (CD3^-^CD49b^+^) in CD45^+^ leukocytes and mCherry^+^ CAR-HMs, CD86^+^ cells and CD206^+^ cells in macrophages from the tumor tissues. (b) DCs (CD80^+^ and CD86^+^) and MDSCs (Arg1^+^) within CD45^+^ leukocytes from the tumor tissues. (c) Ki-67^+^ cells, granzyme B^+^ cells, and IFN-γ^+^ cells in CD4^+^ and CD8^+^ T lymphocytes from the tumor tissues.


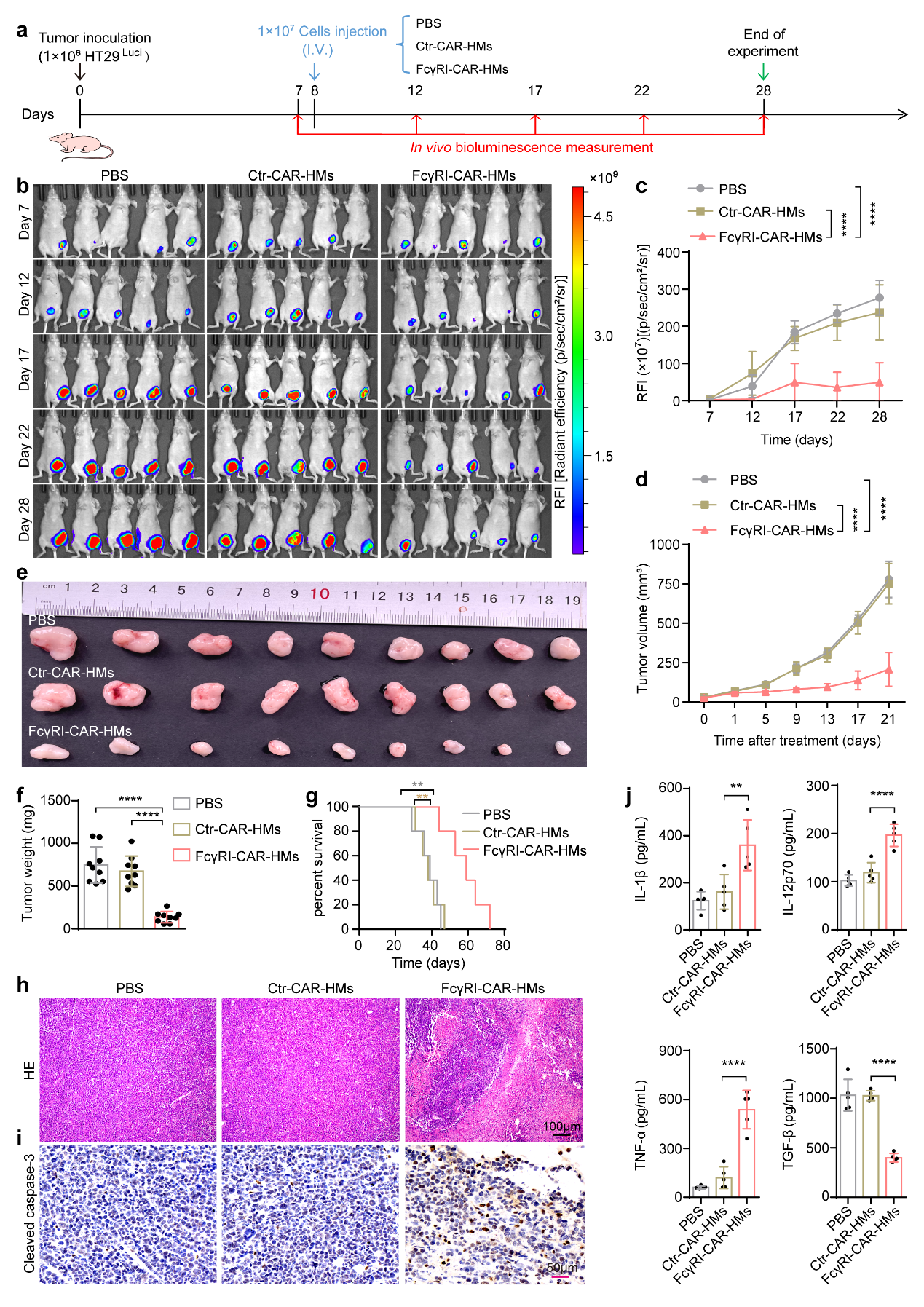


**Supplementary Figure 13. The administration of FcγRI-CAR-HMs effectively inhibited tumor growth in HT29 tumor bearing mice.** (a) Schematic diagram of HT29 tumor bearing mice intravenously injected with different types of CAR-HMs (1×10^7^ cells per mouse) on day 8 post tumor cell inoculation. (b-c) Bioluminescence imaging and related quantified fluorescence intensity of mice with above-mentioned treatments (n = 5 mice per group). (d) Tumor growth curve in mice treated with different CAR-HMs on day 28 post tumor cell inoculation (n = 5 mice per group). (e-f) Tumor images and tumor weights in mice treated with different CAR-HMs on day 28 post tumor cell inoculation (n = 9 mice per group). (g) Survival curves from another batch of mice with the treatment of FcγRI-CAR-HMs or Ctr-CAR-HMs (n = 5 mice per group). Survival data were analyzed by using the log-rank (Mantel-Cox) test. (h-i) Representative H&E and immunohistochemical staining of Cleaved-caspase-3 in HT29 tumor tissues from mice with above mentioned treatments on day 28 post tumor cell inoculation. Scale bars are included in panels (h-i) for reference. (j) Cytokine levels in HT29 tumor tissues from mice treated with different types of CAR-HMs were measured by ELISA on day 28 after tumor cell inoculation (n = 5 mice per group). Data are shown as means ± SD. Statistical analyses were performed using two-way ANOVA test with Sidak's multiple comparisons test for panels (c, d) and one-way ANOVA test with Tukey's multiple comparisons test for panels (f, j). Significance: **P < 0.01, ****P < 0.0001.


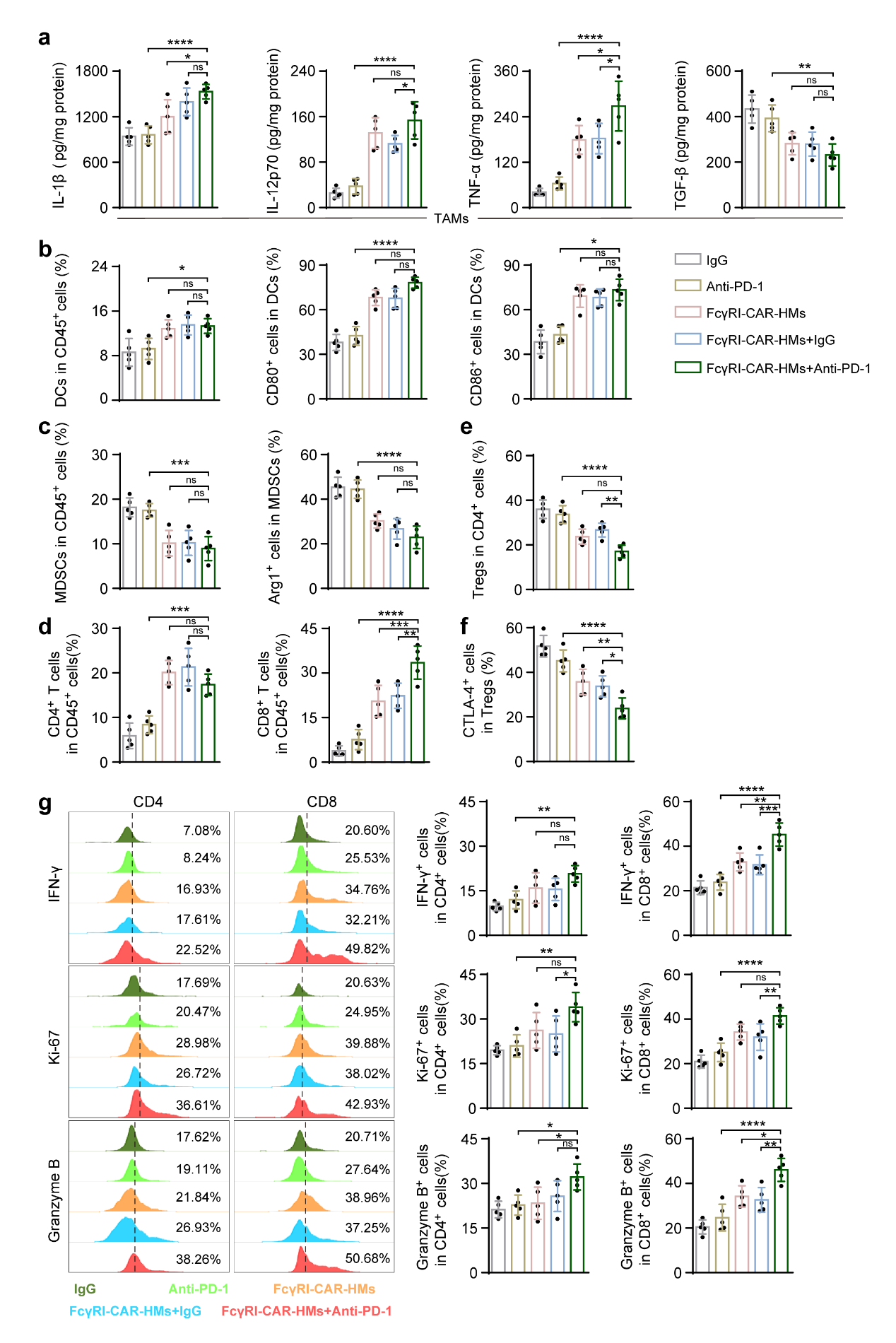


**Supplementary Figure 14. The combined treatment of FcγRI-CAR-HMs and PD-1 antibody induced potent antitumor immunity.** (a) Cytokine levels in TAMs from MC38^CEA^ tumor-bearing mice with for intravenous injection of various CAR-HMs (1×10^7^ cells per mouse) and intraperitoneal injection of anti-PD-1 antibody (10 mg/kg) were measured on day 28 after tumor model establishment. (b-g) The leukocyte suspensions from tumor tissues of mice with above-mentioned treatments were analyzed on day 28 post tumor cell inoculation by flow cytometry. The influence of combined treatment on (b) DCs ratio and activated DCs (CD80^+^ and CD86^+^), (c) ratios of MDSCs and Arg1^+^ MDSCs, (d) CD4^+^ T cells and CD8^+^ T cells ratios, (e-f) ratios of Tregs and CTLA-4^+^ Tregs, (g) T cells activation and proliferation (percentage of IFN-γ^+^, ki-67^+^ and Granzyme B^+^ cells in CD4^+^ T cells and CD8^+^ T cells) was examined. n = 5 mice per group. Data are shown as means ± SD. Statistical analyses were performed using one-way ANOVA test with Tukey's multiple comparisons test for panels (a-g) except panel (b, CD86^+^ DC ratio) and Kruskal-Wallis test with Dunnett's multiple comparisons test for panel (b, CD86^+^ DC ratio). Significance: *P < 0.05, **P < 0.01, ***P < 0.001, ****P < 0.0001, ns, not significant.


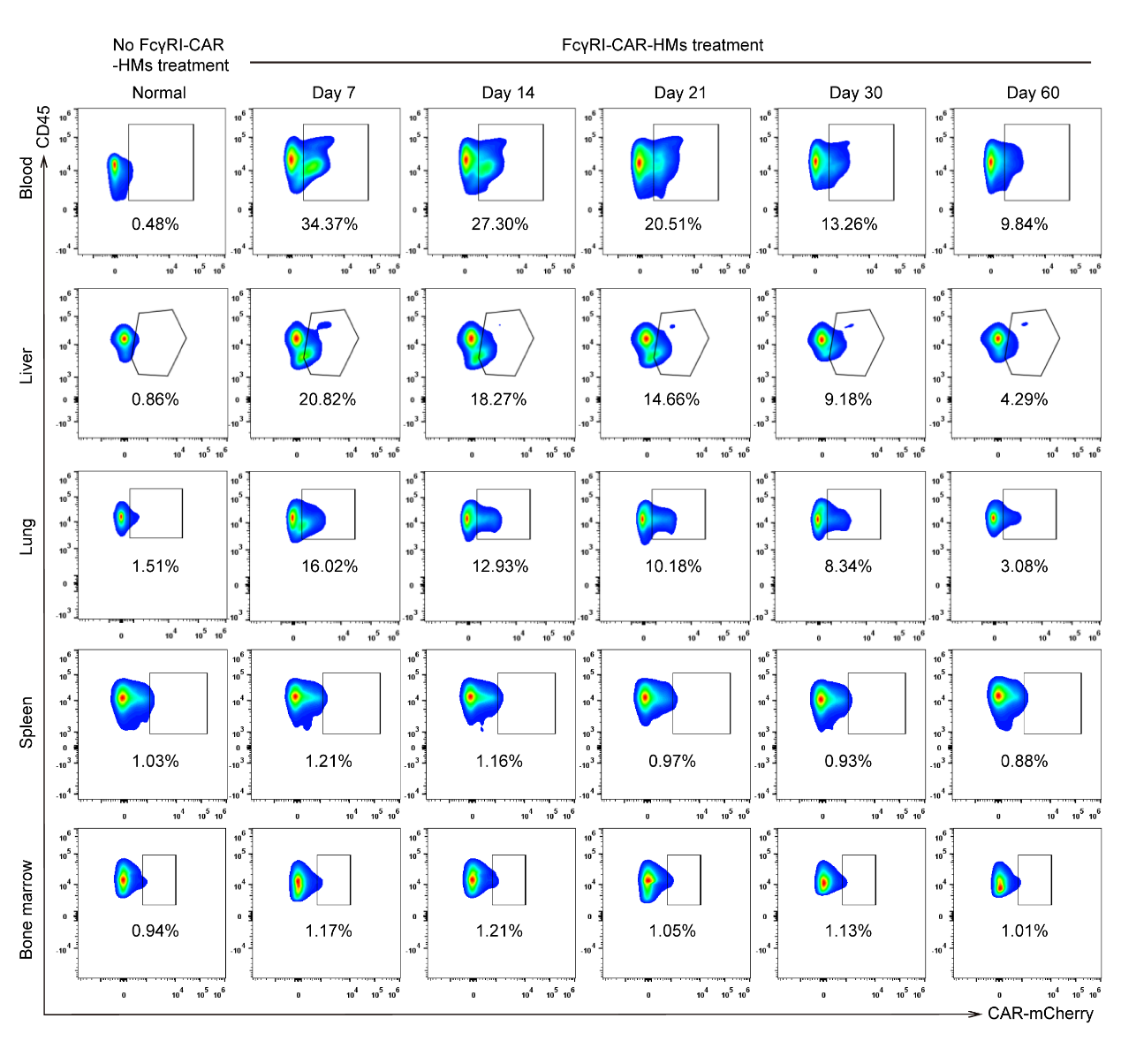


**Supplementary Figure 15. Persistence of FcγRI-CAR-HMs in different organs *in vivo*.** The proportion of FcγRI-CAR-HMs in blood, liver, lung, spleen, and bone marrow at various time points post-in intravenous injection (1×10^7^ cells per mouse) was assessed through flow cytometry analysis. n = 3 mice per group. Representative images are shown.


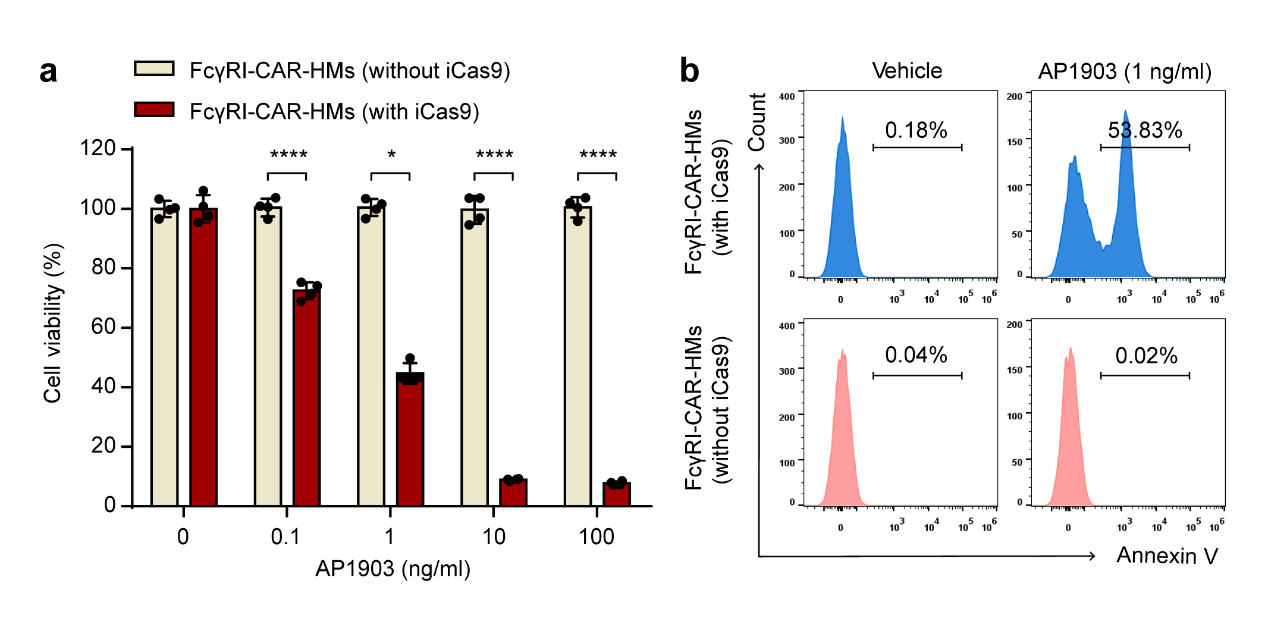


**Supplementary Figure 16. AP1903 induced apoptosis in suicide-switch FcγRI-CAR-HMs.** (a) The viability of FcγRI-CAR-HMs with or without iCas9 treated with different concentrations of AP1903 was assessed using the CCK-8 assay (n = 4 biologically independent samples). (b) Flow cytometry analysis of the apoptotic ratio in FcγRI-CAR-HMs with iCas9 or without iCas9 after a 6 hours exposure to AP1903 (1 ng/mL). Results in panel (b) are representative plots from three biological replicates. Data are presented as means ± SD. Statistical analysis was performed using two-tailed unpaired t test for panel (a: AP1903 = 0.1 ng/mL group), two-tailed unpaired t test with Welch’s correction for panel (a: AP1903 = 10 ng/mL or 100 ng/mL group), and Mann Whitney test for panel (a: AP1903 = 1 ng/mL). Significance: *P < 0.05, ****P < 0.0001.

**Supplementary Tables**

**Supplementary Table 1. The purity of HPCs differentiated-HMs was assessed by flow cytometry. Data are presented as the means ± SD. n = 3 biological replicate.**

| Marker | F4/80^+^CD11b^+^ | CD11c^+^ | Ly6C^+^ | Ly6G^+^ | CD117^+^ |
| --- | --- | --- | --- | --- | --- |
| Positive ratio (%) | 98.3±0.2 | 0.19±0.11 | 0.21±0.06 | 0.37±0.08 | 0.52±0.12 |

**Supplementary Table 2. The number of cells obtained from different types of CAR-HPCs for 10 days. Data are presented as the means ± SD. n = 3 biologically independent sample.**

|  | UTD-HPCs | Ctr-CAR-HPCs | CD3ζ-CAR-HPCs | FcγRI-CAR-HPCs |
| --- | --- | --- | --- | --- |
| Cell number (Day 0, ×10^6^) | 0.1 | 0.1 | 0.1 | 0.1 |
| Cell number (Day 10, ×10^6^) | 5.52±0.64 | 5.83±0.59 | 5.77±0.71 | 5.75±0.62 |

**Supplementary Table 3. The ratios of immune cells in the TME with CAR-HMs treatment.** **Data are presented as the** **means ± SD. n = 5 mice per group.** **Statistical analyses were performed using one-way ANOVA test with Tukey's multiple comparisons test. Significance: *P < 0.05, ****P < 0.0001, ns, not significant.**

|  | PBS | Ctr-CAR-HMs | FcγRI-CAR-HMs | *P* value  (FcγRI-CAR-HMs VS. Ctr-CAR-HMs) |
| --- | --- | --- | --- | --- |
| TAMs (%) | 10.64±0.70 | 12.35±1.67 | 22.39±2.58 | **** |
| T cells (CD3^+^) (%) | 11.91±1.71 | 16.63±1.45 | 28.82±3.39 | **** |
| MDSCs (%) | 14.56±1.90 | 12.66±0.97 | 5.67±0.75 | **** |
| NK cells (%) | 1.67±0.27 | 1.95±0.34 | 6.95±0.51 | **** |
| DCs (%) | 7.79±1.73 | 8.77±2.41 | 12.90±1.82 | * |
| B cells (%) | 7.45±0.97 | 7.18±1.26 | 7.75±1.26 | ns |

**Supplementary Table 4. Primers used in this study, related to Methods.**

| Name of primer | Sequence |
| --- | --- |
| Mouse *β-actin*-Forward | CTACCTCATGAAGATCCTGACC |
| Mouse *β-actin*-Reverse | CACAGCTTCTCTTTGATGTCAC |
| Mouse *Itgax*-Forward  Mouse *Itgax*-Reverse | TCATCACTGATGGGAGAAAACA  CCCCAATTGCATAACGAATGAT |
| Mouse *CD86*-Forward  Mouse *CD86*-Reverse | ACGGAGTCAATGAAGATTTCCT  GATTCGGCTTCTTGTGACATAC |
| Mouse *Ym1*-Forward  Mouse *Ym1*-Reverse  Mouse *Fizz1*-Forward  Mouse *Fizz1*-Reverse  Mouse *Arg1*-Forward  Mouse *Arg1*-Reverse  Mouse *iNOS*-Forward  Mouse *iNOS*-Reverse  Mouse *Ccl5*-Forward  Mouse *Ccl5*-Reverse  Mouse *Ccl2*-Forward  Mouse *Ccl2*-Reverse  Mouse *Il12b*-Forward  Mouse *Il12b*-Reverse  Mouse *Il1a*-Forward  Mouse *Il1a*-Reverse  Mouse *Il1b*-Forward  Mouse *Il1b*-Reverse  Mouse *Il6*-Forward  Mouse *Il6*-Reverse  Mouse *Tnf*-Forward  Mouse *Tnf*-Reverse  Mouse *Ifng*-Forward  Mouse *Ifng*-Reverse  Mouse *Il15*-Forward  Mouse *Il15*-Reverse  Mouse *Il10*-Forward  Mouse *Il10*-Reverse  Mouse *Icam1*-Forward  Mouse *Icam1*-Reverse  Mouse *Icam4*-Forward  Mouse *Icam4*-Reverse  Mouse *Itgb2*-Forward  Mouse *Itgb2*-Reverse  Mouse *Sell*-Forward  Mouse *Sell*-Reverse  Mouse *Cxcl16*-Forward  Mouse *Cxcl16*-Reverse  Mouse *Cxcl15*-Forward  Mouse *Cxcl15*-Reverse  Mouse *Cxcl9*-Forward  Mouse *Cxcl9*-Reverse  Mouse *Cxcl1*-Forward  Mouse *Cxcl1*-Reverse | CAGTGTTCTGGTGAAGGAAATG  ACCCAGACTTGATTACGTCAAT  ATCGTGGAGAATAAGGTCAAGG  TTGACACTAGTGCAAGAGAGAG  CATATCTGCCAAAGACATCGTG  GACATCAAAGCTCAGGTGAATC  TGCATGACACTCTTCACCACAAGG  TCTCTGGGTCCTCTGGTCAAACTC  GTATTTCTACACCAGCAGCAAG  TCTTGAACCCACTTCTTCTCTG  TTTTTGTCACCAAGCTCAAGAG  TTCTGATCTCATTTGGTTCCGA  TGAGAAGTATTCAGTGTCCTGC  CTGTGAGTTCTTCAAAGGCTTC  GTTCCTGACTTGTTTGAAGACC  GTTGGACATCTTTGACGTTTCA  TGTACAAGGAGAACCAAGCAACGAC  TGCCGTCTTTCATTACACAGGACAG  CTCCCAACAGACCTGTCTATAC  CCATTGCACAACTCTTTTCTCA  ATGTCTCAGCCTCTTCTCATTC  GCTTGTCACTCGAATTTTGAGA  ACTGGCAAAAGGATGGTG  GTTGCTGATGGCCTGATT  TCTCCTGGAATTGCAGGTTATT  GCCAGATTCTGCTACATTCTTG  GCTGGACAACATACTGCTAACC  ATTTCCGATAAGGCTTGGCAA  CTGAAAGATGAGCTCGAGAGTG  AAACGAATACACGGTGATGGTA  AAGTACACTCTGCGATGCTATG  GACATTAGCCAAATCTGAACCG  CAGGAATGCACCAAGTACAAAGT  CCTGGTCCAGTGAAGTTCAGC  TGCCCAAAAGCCCTTATTACTA  CTCCTTGGACTTCTTGTTGTTG  CTGGAAGTTGTTCTTGTGATCG  CTGCAACTGGAACCTGATAAAG  CTGTTGGCCCAATTACTAACAG  TCCCGAATTGGAAAGGGAAATA  AATCCCTCAAAGACCTCAAACA  TCCCATTCTTTCATCAGCTTCT  CCGAAGTCATAGCCACACTCAAG  ACCAGACAGGTGCCATCAGAG |
| Mouse *Ccr7*-Forward  Mouse *Ccr7*-Reverse  Mouse *Ccr5*-Forward  Mouse *Ccr5*-Reverse  Mouse *Ccr4*-Forward  Mouse *Ccr4*-Reverse  Mouse *Ccr3*-Forward  Mouse *Ccr3*-Reverse  Mouse *Ccr2*-Forward  Mouse *Ccr2*-Reverse  Mouse *Ccr1*-Forward  Mouse *Ccr1*-Reverse  Mouse *Cxcr2*-Forward  Mouse *Cxcr2*-Reverse  Mouse *Cxcr1*-Forward  Mouse *Cxcr1*-Reverse  Human *β-actin*-Forward  Human *β-actin*-Reverse  Human *CEA*-Forward  Human *CEA*-Reverse  *CAR*-Forward  *CAR*-Reverse | GATGACTACATCGGCGAGAATA  GATGACTACATCGGCGAGAATA  GCTCATCTTTGCCATCATGATT  ATAGATGACAGGGTTTAGGCAG  AAATACAAGAGGCTCAAGTCCA  GATGGCCAGGTATCTGTCTATG  AATCAAGACTGTGGTTGAAAGC  GAGGATCAACACAACCATCATG  GCTCATCTTTGCCATCATGATT  TCATTCCAAGAGTCTCTGTCAC  ATCCTGTTGACGATTGACAGAT  TGATGCCAAAAGTAACAGTTCG  GGGCTGCATCTAAAGTAAATGG  CAGAACACTGCTGTAGAAGGTA  CTAGCCATCGCCGACCTACTC  GCAGCAGGATACCACTGAAGAAG  GCGGGAAATCGTGCGTGAC  GGAAGGAAGGCTGGAAGAG  GATGCTGTGGCCTTCACCTGTG  GTGAGGGTCCTGTTGCCATTGG  CTGTACCTGCAGATGAACAGC  CCGCCAGAAGAAACTGTAACCA |

**Supplementary Table 5. Antibodies used in this study, related to Methods.**

| Antibody | Catalogue No. | Manufacturer | Concentration | Application |
| --- | --- | --- | --- | --- |
| Hoxb8 | sc-517156 | Santa Cruz Biotechnology | 1:1000 | WB |
| Anti-Syk | ab40781 | Abcam | 1:1000 | WB |
| Anti-Syk (phospho Y352) +ZAP70 (phospho Y319) | ab300398 | Abcam | 1:1000 | WB |
| Bcl-2 | ab182858 | Abcam | 1:2000 | WB |
| Bax | ab32503 | Abcam | 1:1000 | WB |
| NF-κB p65 (D14E12) XP® Rabbit mAb | 8242S | Cell Signaling Technology | 1:1000 | WB |
| Phospho-NF-κB p65 (Ser536) (93H1) Rabbit mAb | 3033S | Cell Signaling Technology | 1:1000 | WB |
| Akt | 9272S | Cell Signaling Technology | 1:1000 | WB |
| Phospho-Akt (Ser473) (D9E) XP® Rabbit mAb | 4060T | Cell Signaling Technology | 1:2000 | WB |
| PARP (Asp214) (D6X6X) Rabbit mAb | 94885S | Cell Signaling Technology | 1:1000 | WB |
| Caspase-3 | 9662S | Cell Signaling Technology | 1:1000 | WB |
| Cleaved-caspase-3 (Asp175) (5A1E) Rabbit mAb | 9654S | Cell Signaling Technology | 1:1000 | WB |
| Anti-Integrin beta 1 | ab179471 | Abcam | 1:2000 | WB |
| Anti-LFA | ab13219 | Abcam | 1:1000 | WB |
| GAPDH | HRP-60004 | Proteintech | 1:10000 | WB |
| CD8 | 98941S | Cell Signaling Technology | 1:200 | IHC |
| Perforin | 31647S | Cell Signaling Technology | 1:100 | IHC |
| Granzyme B | 46890S | Cell Signaling Technology | 1:50 | IHC |
| Cleaved-caspase-3 | 9579S | Cell Signaling Technology | 1:250 | IHC |
| APC anti-mouse CD117 | 105811 | BioLegend | 1:20 | FC |
| Brilliant Violet 711 anti-mouse F4/80 | 123147 | BioLegend | 1:50 | FC |
| APC anti-mouse/human CD11b | 101212 | BioLegend | 1:100 | FC |
| Phycoerythrin anti-mouse CD11c | 117307 | BioLegend | 1:100 | FC |
| BV421 anti-mouse Ly6G | 127627 | BioLegend | 1:50 | FC |
| PE/Cyanine7 anti-mouse Ly6C | 128017 | BioLegend | 1:200 | FC |
| BV421 anti-mouse CD86 | 105123 | BioLegend | 1:50 | FC |
| APC anti-human CEA | 342308 | BioLegend | 1:20 | FC |
| anti-mouse CD16/32 | 101320 | BioLegend | 1:50 | FC |
| PE/Cyanine7 anti-mouse CD45 | 157205 | BioLegend | 1:100 | FC |
| PE anti-mouse CD45 | 147711 | BioLegend | 1:100 | FC |
| PE/Cyanine7 anti-mouse CD206 | 141720 | BioLegend | 1:100 | FC |
| PE anti-mouse Gr-1 | 108407 | BioLegend | 1:100 | FC |
| BV605 anti-mouse CD3 | 100237 | BioLegend | 1:20 | FC |
| BV421 anti-mouse CD4 | 116023 | BioLegend | 1:200 | FC |
| APC anti-mouse CD8 | 100712 | BioLegend | 1:100 | FC |
| PE/Cyanine7 anti-mouse CD49b | 108921 | BioLegend | 1:50 | FC |
| BV711 anti-mouse CD19 | 115555 | BioLegend | 1:100 | FC |
| BV605 anti-mouse CD11c | 117333 | BioLegend | 1:20 | FC |
| PE anti-mouse Arginase 1 | 165803 | BioLegend | 1:200 | FC |
| BV711 anti-mouse CD80 | 104743 | BioLegend | 1:50 | FC |
| APC anti-mouse CD197 (CCR7) | 120107 | BioLegend | 1:20 | FC |
| PE anti-mouse CD192 (CCR2) | 150609 | BioLegend | 1:50 | FC |
| PE anti-mouse CD152 | 106305 | BioLegend | 1:20 | FC |
| BV421 anti-mouse Foxp3 | 126419 | BioLegend | 1:50 | FC |
| APC anti-mouse CD25 | 113709 | BioLegend | 1:50 | FC |
| BV711 anti-mouse IFN-γ | 505836 | BioLegend | 1:20 | FC |
| APC anti-mouse Ki-67 | 652406 | BioLegend | 1:100 | FC |
| PE anti-mouse Granzyme B | 372208 | BioLegend | 1:20 | FC |
| Anti-mouse IgG | BE0085 | Bio X cell | 10mg/kg per mouse | *In vivo* |
| Anti-mouse PD-1 | BE0146 | Bio X cell | 10mg/kg per mouse | *In vivo* |
